# Naïve pluripotency and genomic stability are coordinated in embryonic stem cells by a novel pluripotency regulator ZFP998

**DOI:** 10.1093/nar/gkag546

**Published:** 2026-05-27

**Authors:** Min Tang, Lin Wang, Jia-wen Liu, Wan-huan Zhong, Han Wu, Wen-hui Nie, Guo-meng Li, Wei-dao Zhang, Hu Zhou, Jing Gao, Ping Zheng

**Affiliations:** State Key Laboratory of Genetic Evolution & Animal Models, Kunming Institute of Zoology, Chinese Academy of Sciences, Kunming 650201, China; Key Laboratory of Animal Models and Human Disease Mechanisms of Yunnan Province, Kunming Institute of Zoology, Chinese Academy of Sciences, Kunming, Yunnan 650201, China; State Key Laboratory of Genetic Evolution & Animal Models, Kunming Institute of Zoology, Chinese Academy of Sciences, Kunming 650201, China; Key Laboratory of Animal Models and Human Disease Mechanisms of Yunnan Province, Kunming Institute of Zoology, Chinese Academy of Sciences, Kunming, Yunnan 650201, China; University of Chinese Academy of Sciences, Beijing 101408, China; University of Chinese Academy of Sciences, Beijing 101408, China; University of Chinese Academy of Sciences, Beijing 101408, China; State Key Laboratory of Genetic Evolution & Animal Models, Kunming Institute of Zoology, Chinese Academy of Sciences, Kunming 650201, China; University of Chinese Academy of Sciences, Beijing 101408, China; State Key Laboratory of Genetic Evolution & Animal Models, Kunming Institute of Zoology, Chinese Academy of Sciences, Kunming 650201, China; Key Laboratory of Animal Models and Human Disease Mechanisms of Yunnan Province, Kunming Institute of Zoology, Chinese Academy of Sciences, Kunming, Yunnan 650201, China; Department of Analytical Chemistry and CAS Key Laboratory of Receptor Research, Shanghai Institute of Materia Medica, Chinese Academy of Sciences, Shanghai 201203, China; Department of Analytical Chemistry and CAS Key Laboratory of Receptor Research, Shanghai Institute of Materia Medica, Chinese Academy of Sciences, Shanghai 201203, China; State Key Laboratory of Genetic Evolution & Animal Models, Kunming Institute of Zoology, Chinese Academy of Sciences, Kunming 650201, China; Key Laboratory of Animal Models and Human Disease Mechanisms of Yunnan Province, Kunming Institute of Zoology, Chinese Academy of Sciences, Kunming, Yunnan 650201, China; KIZ/CUHK Joint Laboratory of Bioresources and Molecular Research in Common Diseases, Kunming Institute of Zoology, Chinese Academy of Sciences, Kunming, Yunnan 650201, China

## Abstract

Efficacy and safety are key objectives in generating high-quality pluripotent stem cells (PSCs). While naïve human PSCs (hPSCs) harbor an unstable genome limiting their broad applications, mouse embryonic stem cells (mESCs) uniquely exhibit both robust pluripotency and high genomic stability. Understanding whether and how these two attributes are co-regulated in mESCs could provide critical insights for producing safe and authentic hPSCs. Here, we reveal that the coordination of naïve pluripotency and genomic stability in mESCs is governed by a novel core pluripotency regulator, ZFP998. ZFP998 binds to promoters and enhancers of key ESC-identity genes, as well as to numerous DNA damage response and repair genes, thereby regulating their expression. Depletion of *Zfp998* leads to the loss of naïve pluripotency and induces severe genomic instability. Conversely, overexpression of ZFP998 is sufficient to reprogram epiblast stem cells back to a naïve pluripotent state. Importantly, ectopic expression of ZFP998 in hESCs enhances both pluripotency and genomic stability. These findings suggest that this coupled regulatory mechanism is conserved in humans and provide a promising new strategy for generating safe, naïve hPSCs.

## Graphical Abstract

**Graphical Abstract:**
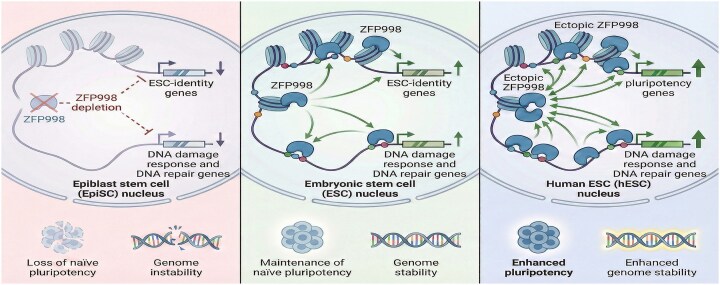

## Introduction

Primates (including humans and monkeys) ESCs derived from pre-implantation embryos closely resemble epiblast cells—rather than the inner cell mass (ICM)—in terms of transcriptomic and methylome profiles. They are therefore considered to be in a “primed” state of pluripotency and exhibit limited developmental potential [1]. Over the past decade, numerous culture conditions have been developed to convert human ESCs (hESCs) from the primed to a “naïve” state in order to enhance their developmental capacity [2, 3]. Although these conditions successfully induce naïve pluripotency features, nearly all resulting cell lines rapidly develop DNA hypomethylation and confounding chromosomal abnormalities within short passages [3], limiting their broader application. Consequently, generating and expanding authentic human pluripotent stem cells (hPSCs) that exhibit full pluripotency along with high epigenetic and genomic stability remains a significant challenge.

In contrast, mouse ESCs (mESCs)—derived directly from the ICM—reside in a naïve pluripotent state. They can be expanded in culture while maintaining robust pluripotency and superior genomic stability in the presence of serum or KnockOut Serum Replacement (KSR), leukemia inhibitory factor (LIF), and feeder condition, rather than under 2i/LIF, which can compromise developmental potential, induce DNA hypomethylation, and cause chromosomal abnormalities [4–10]. Understanding how mESCs simultaneously sustain both authentic pluripotency and genomic integrity could provide crucial insights for improving hPSC quality. A recent comparative study between naïve mESCs and primed mouse epiblast stem cells (EpiSCs) revealed a strong link between pluripotent state and genomic stability: a decline in pluripotency was accompanied by reduced genomic stability. Specifically, mouse EpiSCs displayed heightened epigenetic and genomic instability, including increased copy number variation, telomere fragility, and aberrant retrotransposon activity. These defects were associated with impaired DNA recombination and repair, compromised alternative lengthening of telomeres, and downregulation of numerous genes involved in DNA replication, double-strand break (DSB) repair, mismatch repair, nucleotide excision repair, and telomere maintenance [5]. Notably, 62 DNA damage response (DDR) genes were significantly downregulated (fold change ≥2) in EpiSCs at passage 5 compared to naïve mESCs [5]. These findings raise an intriguing question: how do mESCs effectively couple pluripotency with genomic stability?

In this study, we address this question by identifying ZFP998 as a central regulator that orchestrates the simultaneous maintenance of naïve pluripotency and genomic stability. ZFP998 not only acts as a novel core regulator of naïve pluripotency but also directly controls the expression of multiple DDR genes, thereby coordinating the maintenance of both naïve pluripotency and genomic stability. Importantly, ectopic expression of ZFP998 in H9 hESCs enhances both pluripotency and genomic stability, suggesting that this coupling mechanism is conserved in humans.

## Materials and methods

### Cell culture

Mouse embryonic fibroblasts (MEFs) were isolated from CD1 mouse embryos at embryonic day 13.5 [11]. The MEFs were cultured in DMEM medium (Gibco, 11965) supplemented with 10% fetal bovine serum (FBS; Gibco, 10099141C). The mESCs used in this study were generated in-house [12] and maintained on mitomycin C-treated MEF feeders in DMEM/F12 medium containing 20% Knockout serum replacement (Gibco, 10828028), 2 mM L-glutamine (Sigma, G8540), 1 mM sodium pyruvate (Gibco, 11360070), 0.1 mM β-mercaptoethanol (Sigma, M7522), 1% non-essential amino acids (Gibco, 11140-035), and 1000 units/ml mouse LIF (Millipore, ESG1107).

Mouse EpiSCs were kindly provided by Professor Jiekai Chen (Guangzhou Institutes of Biomedicine and Health, Chinese Academy of Sciences). These cells were derived from embryonic day 5.5 embryos obtained by crossing homozygous POU5F1-GFP transgenic mice (CBA/CaJ × C57BL/6J) with 129/Sv female mice. EpiSCs were maintained in a feeder-free manner on FBS-coated dishes in FA medium, which consisted of N2B27 base medium supplemented with 15 ng/ml bFGF (Merck Millipore, GF003) and 20 ng/ml activin A (MCE, HY-P70311). The N2B27 medium consisted of a 1:1 mixture of DMEM/F12 (Gibco, 11320033) and Neurobasal medium (Gibco, 21103049), further supplemented with 0.5 × N2 (Gibco, 17502048), 0.5 × B27 (Gibco, 17504044), non-essential amino acids (Gibco, 11140050), GlutaMAX (Gibco, 35050061), 0.1 mM β-mercaptoethanol (Thermo Fisher, 21985023), and 1 μM XAV939 (Sigma, X3004).

H9 hESCs were cultured in Essential 8 medium on Matrigel-coated dishes (Corning, 354277). All cells were maintained at 37°C in a humidified incubator with 5% CO_2_.

For the resetting of EpiSCs to naïve PSCs, EpiSCs were dissociated into single cells using Accutase (Sigma, A6964) and seeded at a density of ~30 000 cells per well on feeder-coated 6-well plates in N2B27-FA medium supplemented with 5 μM Y27632 (MCE, HY-10583). After 24 h, the medium was switched to N2B27-2i/LIF, and the cells were cultured for an additional 3 days with daily medium changes.

### CRISPR–Cas9 screen for genome stability regulators in mESCs

To identify regulators of genome stability in mESCs, a customized CRISPR–Cas9 knockout library was designed to target mESC-specific genes. Specifically, mESC-specific genes were defined by comparative transcriptomic analysis against multiple differentiated tissues from the ENCODE project, including stomach, intestine, lung, heart, liver, spleen, kidney, and colon. For quality control and benchmarking, well-characterized DDR genes were selected from published studies as positive controls, while genes associated with cell viability were included as internal controls for CRISPR screening performance.

Single-guide RNAs (sgRNAs) were designed using the CRISPR-FOCUS online platform (http://cistrome.org/crispr-focus/) [13], with 10 independent sgRNAs designed per gene to ensure robust targeting efficiency. The oligonucleotide pool containing all sgRNA sequences was synthesized by Genewiz (Suzhou, China) and cloned into the lentiGuide-Puro lentiviral vector using the EasyGeno Assembly kit (VI201; TIANGEN). The constructed sgRNA library pool was validated by next-generation sequencing to ensure proper representation and minimal bias.

For pooled screening, Cas9-stably expressing mESCs were infected with the sgRNA library lentivirus at a low multiplicity of infection (MOI = 0.3) to minimize multiple integrations. Infected cells were selected with puromycin for 3 days to eliminate uninfected cells and achieve efficient gene knockout before chemical treatment.

To determine appropriate treatment conditions, inhibitory concentrations (IC) of each DNA-damaging agent were titrated in mESCs, and the IC_20_ concentration was chosen to minimize acute cytotoxicity while retaining biological effects. Library-infected mESCs were then treated with each DNA-damaging compound at its IC_20_ for 7 days, with 0.1% dimethyl sulfoxide used as a vehicle control. After treatment, cells were harvested, and genomic DNA was extracted for sgRNA amplification.

sgRNA sequences were polymerase chain reaction (PCR)-amplified using a previously established protocol [14] and subjected to high-throughput sequencing on an Illumina platform. Raw sequencing reads were trimmed and filtered using fastx_trimmer from the FASTX-Toolkit. Read counts for individual sgRNAs were quantified using the MAGeCK (version 2.2.1) computational framework [15]. Genes with statistically significant changes in sgRNA abundance were identified using the test subcommand of MAGeCK.

### Construction of plasmids and establishment of ESC lines

Short hairpin RNA (shRNA) sequences were designed using an online tool (https://rnaidesigner.thermofisher.com/rnaiexpress/design.do). The shRNAs were synthesized and cloned into the pLKO.1 plasmid. The codon-optimized coding sequence of *Zfp998* (with an N-terminal 3xFLAG tag, amino acid sequence unchanged) was synthesized by Sino Biological Inc. and inserted into either the pTOMO-IRES-EGFP lentiviral expression vector or pPB-CAG-TetOn3G-IRES-Puro transposon vector (a gift from Professor Jie Na, Tsinghua University). Lentivirus for shRNA or 3xFLAG-tagged ZFP998 expression was produced in 293T cells using the psPAX2 and pMD2.G packaging plasmids. mESCs were transduced with the corresponding lentivirus. Cells transduced with shRNA constructs were selected with 0.5 μg/ml puromycin for 72 h, while those expressing FLAG-tagged ZFP998 were sorted based on green fluorescence.

To generate a *Zfp998* knockout mESC line, we designed two sgRNAs targeting the first intron and within the body of exon 4 of the long transcript of *Zfp998*, aiming to delete an ~4.0 kb genomic fragment covering exons 2–4. This deletion removes 99.8% of the coding sequence (1392 bp). Each sgRNA was cloned into a separate px330 plasmid. Both plasmids were co-transfected into wild-type (WT) mESCs using Lipofectamine 2000. Single-cell clones were picked and screened by genomic PCR to obtain homozygous *Zfp998* knockout cell lines.

For H9 hESCs transfection, cells were electroporated with the PiggyBac transposon vector and hyperPB messenger RNA (mRNA) using the Neon system. Transfected H9 hESCs were selected with 0.5 μg/ml puromycin. Sequences for shRNAs, primers, and sgRNAs are provided in Supplemental Data S1.

### Generation of chimeric embryos

To evaluate the germline transmission potential of reset PSCs (rPSCs), cells were transduced with a lentiviral vector expressing red fluorescent protein (RFP). RFP-positive rPSCs were then microinjected into eight-cell embryos collected from C57BL/6 mice. After 24 h of *in vitro* culture in KSOM medium, the resulting chimeric blastocysts were surgically transferred into the uterine horns of pseudopregnant ICR recipient females (2–3 months old) at 2.5 days post coitum. Embryos were collected at embryonic day 13.5 (E13.5). All animal care and experimental procedures were approved by the Institutional Animal Care and Use Committee of the Kunming Institute of Zoology, Chinese Academy of Sciences.

### Immunoprecipitation, immunoblotting, and immunofluorescence staining

For immunoprecipitation, cells were lysed in RIPA lysis buffer (Beyotime, P0013J) containing 1× protease inhibitor cocktail (Beyotime, P1006). The lysates were incubated overnight at 4°C with primary antibody and Protein G Dynabeads (Thermo, 88847). The supernatant was then removed, and the beads were washed three times with RIPA buffer. Subsequently, the beads were boiled at 100°C in 2 × sodium dodecyl sulfate (SDS) protein loading buffer for 5 min. The eluted proteins were analyzed by mass spectrometry (MS) or immunoblotting.

Immunoblotting and immunofluorescence staining were performed according to standard protocols. For immunoblotting, protein extracts were separated by SDS–polyacrylamide gel electrophoresis, and transferred onto polyvinylidene fluoride membranes. After blocking with 5% bovine serum albumin (BSA) for 1 h at room temperature, the membranes were incubated with primary and secondary antibodies. Protein bands were detected using ECL reagent (Beyotime, P0018M) and imaged with a ProteinSimple FluorChem system (Fluorchem M, FM0561).

For immunofluorescence staining, cells were fixed with 4% paraformaldehyde (PFA) in PBS for 15 min at room temperature, permeabilized with 0.25% Triton X-100 in PBS for 5 min, and blocked with 1% BSA for 1 h. Then, the cells were incubated with primary antibodies followed by fluorophore-conjugated secondary antibodies. Nuclei were counterstained with 4′,6-diamidino-2-phenylindole (DAPI) for 10 min. Images were acquired using an Olympus FV1000 confocal microscope (Japan). All antibody information is provided in Supplemental Data S2.

### Cell proliferation assay

Cell proliferation was assessed using the Cell Counting Kit-8 (CCK-8; FuHeng, FH200-10) according to the manufacturer’s instructions. Briefly, cells were seeded in triplicate into 96-well plates at a density of 2000 cells per well. At the indicated time points, 10 μl of CCK-8 reagent was added directly to each well containing 100 μl of culture medium. The plates were incubated at 37°C for 2 h, and the absorbance at 450 nm was measured using a microplate reader. The absorbance value is proportional to the number of viable cells.

### Quantitative RT-PCR

Total RNA (1 µg) was reverse-transcribed using the PrimeScript™ RT Reagent Kit with gDNA Eraser (Perfect Real Time; Takara, RR047A). Quantitative real-time PCR was performed using the TB Green™ Premix Ex Taq™ II kit (Takara, RR820A) in a CFX96^TM^ Real-Time System (Bio-Rad, CFX96 Touch). Each reaction contained 10 μl of TB Green Premix, 0.2 µM of each primer, and 2 μl of 1:10 diluted cDNA in a final volume of 20 μl. The thermal cycling protocol consisted of an initial denaturation at 95°C for 30 s, followed by 40 cycles of 95°C for 5 s and 60°C for 30 s. A melting curve analysis was performed to verify amplification specificity. *Actb* was used as the internal reference gene for normalization, and relative expression was quantified using the 2^−ΔΔCt^ method. No-template controls and no-reverse-transcription controls were included in each run. All reactions were performed in triplicate (technical replicates), and three independent biological replicates (*n* = 3) were analyzed. Primer sequences are listed in Supplementary Data S1.

### Telomere *in situ* hybridization

Telomere *in situ* hybridization was carried out as previously described [16, 17]. In brief, cultured cells were fixed with 4% PFA for 10 min, dehydrated through a graded ethanol series (70%, 85%, and 100%; 5 min each), and hybridized overnight at 37°C with 0.05 pmol/l of a Cy3-labeled peptide telomeric PNA probe (sequence: T_2_AG_3_) in hybridization buffer (20 mmol/l Na_2_HPO_4_, 20 mmol/l Tris, 60% formamide, 2 × SSC, pH = 7.4). Nuclei were counterstained with DAPI. Images were acquired using an Olympus FV1000 confocal microscope (Japan). Three independent experiments were performed, with at least 50 cells analyzed per group.

### Karyotyping

Cells were treated with 120 ng/ml KARYOMAX colcemid (Gibco, 15212-012) for 2 h, trypsinized with 0.05% trypsin–ethylenediaminetetraacetic acid (EDTA; Invitrogen, 25200072), resuspended in 0.075 M KCl, and incubated at 37°C for 20 min. After fixation in methanol/glacial acetic acid (3:1) for 10 min, cells were dropped onto slides, air-dried, and stained with Giemsa solution (Gibco, 10092013). At least 50 metaphase spreads were analyzed across three independent replicates.

### DNA fiber assay

The DNA fiber assay was performed following a published protocol [18]. Briefly, cells were pulse-labeled with 50 µM 5-iodo-2′-deoxyuridine (IdU; Sigma, I7125) for 30 min, followed by treatment with 4 mM hydroxyurea (HU; Selleck, S1896) for 0 or 4 h, and then labeled with 50 µM 5-chloro-2′-deoxyuridine (CldU; Sigma, C6891) for 30 or 90 min. Cells were harvested and resuspended in PBS. A total of 2.5 μl cell suspension (~3000 cells) was mixed with 7.5 μl lysis buffer (50 mM EDTA, 0.5% SDS, 200 mM Tris–HCl, pH 7.5) on a glass slide, tilted to 15°, and air-dried. Slides were fixed in methanol: acetic acid (3:1), denatured in 2.5 M HCl at 37°C for 30 min, and immunostained with rat anti-BrdU/CldU (Abcam, ab6326) and mouse anti-IdU (BD, 347580) antibodies at 4°C overnight. After incubation with Alexa Fluor-conjugated secondary antibodies (AlexaFluor Cy3-coupled goat anti-rat and Alexa Fluor 488-coupled goat anti-mouse) for 1 h, DNA fibers were imaged using an Olympus FV1000 confocal microscope (Japan).

### Neutral comet assay

The neutral comet assay was conducted as described [64]. Briefly, 10 μl of cell suspension (~100 000 cells/ml) was mixed with 70 μl 0.8% low-melting agarose (Sangon Biotech, A600015-0025) and layered onto slides pre-coated with 0.8% agarose. Slides were immersed in neutral lysis solution (2.5 M NaCl, 100 mM Na_2_ EDTA, 10 mM Tris, 1% N-lauroylsarcosine, 1% Triton X-100, pH 9.5) for 60 min, and equilibrated in electrophoresis buffer (300 mM sodium acetate, 100 mM Tris, pH 8.3) for 20 min at room temperature, and electrophoresed at 80 mA for 30 min. After fixation in ethanol and staining with DAPI (10 ng/ml), comets were analyzed using Komet 7 software (Andor Technology).

### Micronucleus analysis

Cells were cultured on Matrigel-coated glass coverslips, fixed with 4% PFA for 15 min at room temperature, and stained with DAPI. Micronuclei were identified according to established cytogenetic criteria as small, round, DNA-containing bodies within the cytoplasm, visibly separated from the main nucleus, sharing the same focal plane, and measuring less than one-third the diameter of the main nucleus. Only intact cells with clear nuclear boundaries were scored. The assay was performed following the standard cytokinesis-block micronucleus cytome assay protocol described previously [19]. At least 1000 cells per group were analyzed across three independent replicates.

### Laser micro-irradiation

Cells were seeded in Matrigel-coated glass-bottom dishes and incubated with 10 μM EdU for 30 min to identify S-phase cells. DNA lesions were induced by a 405-nm laser for 15 s using an Olympus FV1000 confocal microscope. After recovery for 1.5 h in the incubator at 37°C, cells were washed once with PBS, fixed in 4% PFA, and processed for immunofluorescence staining. At least 50 cells were analyzed per experiment over three replicates.

### RNA sequencing and data analyses

Total RNA was extracted from the samples using TRNzol Universal (Tiangen, DP424) and reverse-transcribed into complementary DNA (cDNA) using the TruSeqTM RNA Sample Preparation Kit (Illumina). Paired-end 150-bp RNA sequencing was performed on a NovaSeq 6000 (Illumina) at Novogene. Clean reads were aligned to the mouse genome (mm10) or human genome (hg19) using TopHat2 (Version 2.1.1) software with the Refseq annotation. Gene expression levels were calculated as FPKM (Fragments Per Kilobase Million) using Cufflinks (version 2.2.1). Differential gene expression (DEG) analysis was performed by Cuffdiff (version 2.2.1), and genes with a *P-*value <0.05, *q-*value <0.05, and a fold change >2 or >1.5 were considered as DEGs. Heatmaps were generated using the “ComplexHeatmap” or “gplots” *R* package. Functional enrichment analysis for Gene Ontology (GO) terms and Kyoto Encyclopedia of Genes and Genomes pathways was conducted using DAVID (http://david.abcc.ncifcrf.gov/).

### CUT&Tag and data analysis

The Cleavage Under Targets and Tagmentation (CUT&Tag) assay was performed using the Hyperactive Universal CUT&Tag Assay Kit for Illumina (Vazyme, TD903) according to the manufacturer’s protocol. Briefly, the cells (10^5^ cells) were harvested into an eight-strip tube containing activated ConA Beads and primary antibody. The mixtures were incubated overnight at 4°C, followed by incubation with the secondary antibodies for 60 min at room temperature, and then with pA/G-Tnp for 1 h at room temperature. After chromatin fragmentation, DNA was extracted and used for library construction with the TruePrep Index Kit V2 for Illumina (Vazyme, TD202).

Raw sequencing reads were processed with Trim_galore (version 0.6.7) to remove adapter sequences and low-quality bases using the following parameters: -j 8 -q 25 --phred33 --length 30 -e 0.1 --stringency 4. The cleaned reads were aligned to the mouse genome (mm10) or human genome (hg19) reference genome using Bowtie2 (version 2.2.5) with the parameters “-X 2000” in addition to default settings. Mitochondrial DNA reads were removed, and PCR duplicates were eliminated using Samtools. Peak calling was performed with MACS2 (version 2.2.5) using the arguments “--nomodel -f BAM -q 0.01.” Signal tracks were generated from aligned reads using the bamCoverage function in deepTools (version 3.5.1) and normalized by reads per kilobase per million mapped reads (RPKM). For histone modification data, normalization was carried out using ChIPseqSpikeInFree (1.2.4). Peak annotation was performed with ChIPseeker (1.34.1), and enrichment of ZFP998 binding motifs was analyzed using HOMER (version 4.11).

### Mass spectrometry data preprocessing

Intensity values of zero were treated as missing values and converted to NA. Proteins with fewer than two valid intensity values in both groups (FLAG-ZFP998-expressing mESCs and WT mESCs) were excluded from subsequent analyses. The remaining proteins were subjected to log_2_ transformation and quantile normalization using the normalizeBetweenArrays function with the parameter method = “quantile” in R. Missing values were imputed via random sampling from a normal distribution—with the mean shifted 1.8 standard deviations lower and the standard deviation set to 0.3 times the original standard deviation of each sample (Perseus-style imputation)—to simulate signals of low-abundance proteins.

The limma package (version 3.60.6) with empirical Bayes moderation (eBayes function with trend = TRUE) was employed as the primary statistical method. A linear model was fitted using the lmFit function with a design matrix of ~ group, where “group” was a factor with WT mESCs as the baseline level and FLAG-ZFP998-expressing mESCs as the comparison group. The empirical Bayes method moderates standard errors toward a common value, borrowing information across all proteins to enhance statistical power in experiments with limited biological replicates (*n* = 3 per group).

Two-tailed independent Student’s *t*-tests were performed to calculate *P*-values. Multiple testing correction was applied using the Benjamini-Hochberg procedure with a false discovery rate (FDR) of 0.05 to control for Type I errors. Proteins were considered significantly differentially expressed if they met two criteria: an absolute log_2_-fold change (|log2FC|) greater than 1 (corresponding to a >2-fold change in protein abundance) and an adjusted *P*-value (FDR-adjusted) <0.05.

Volcano plots were generated using the ggplot2 package (version 3.5.2). Genes of interest (P300, POU5F1, SOX2, LIN28A, ESRRB, KLF4, DDX5, TRIM28, DDX17, ZFP998) were highlighted and labeled using the ggrepel package (version 0.9.6) to avoid label overlap.

### ATAC-seq and data analysis

Assay for transposase-accessible chromatin with high-throughput sequencing (ATAC-seq) was performed using the Hyperactive ATAC-Seq Library Prep Kit for Illumina (Vazyme, TD711) according to the manufacturer’s instructions. Briefly, ~10^5^ viable cells (>90% viability) were collected, washed twice with cold TW buffer, and lysed in pre-chilled lysis buffer. After centrifugation, the nuclei were resuspended in Tagmentation Mix (containing Tn5 transposase) and incubated at 37°C for 30 min to fragment open chromatin. The reaction was terminated with stop buffer, and DNA was extracted using ATAC DNA extract beads. The eluted DNA was amplified via PCR with index primers (Vazyme, TD202). The libraries were purified using ATAC DNA clean beads through a two-step size selection, followed by washing with ethanol, air-drying, and elution in 22 μl of H_2_O.

Raw ATAC-seq reads were processed with Trim_galore (version 0.6.10) to remove adapters and low-quality sequences using the following parameters: -j 3 -q 25 --phred33 --length 25 -e 0.1 --stringency 4. The cleaned reads were aligned to the mouse mm10 genome using Bowtie2 (version 2.4.1) with the parameters “-X 2000.” Mitochondrial DNA reads were filtered out from the SAM files using the “grep –v chrM.” The SAM files were then converted to BAM format, and duplicate reads were removed using Samtools. For visualization, BAM files were converted to Bigwig format using deepTools (version 3.5.4) [20] with RPKM normalization.

Peak calling was performed with MACS2 (version 2.2.5) [21] under the following settings: “--nomodel -f BAM -q 0.01.” Peaks from each sample were merged into a unified set using the BEDTools merge function. Annotation of ATAC-seq peaks was conducted using ChIPseeker [22]. The RPKM-normalized BigWig files were used to compute peak values via the deepTools multiBigwigSummary function.

### Statistical analysis

Data were analyzed using GraphPad Prism 8 (GraphPad Software, La Jolla, CA, USA) through two-tailed Student’s *t*-test. *P* < 0.05 was considered significant. All data are provided as the mean ± SEM. All experiments were repeated at least three times.

## Results

### Identification of ZFP998 in maintaining naïve pluripotency and genomic stability in mESCs

**Figure 1.**
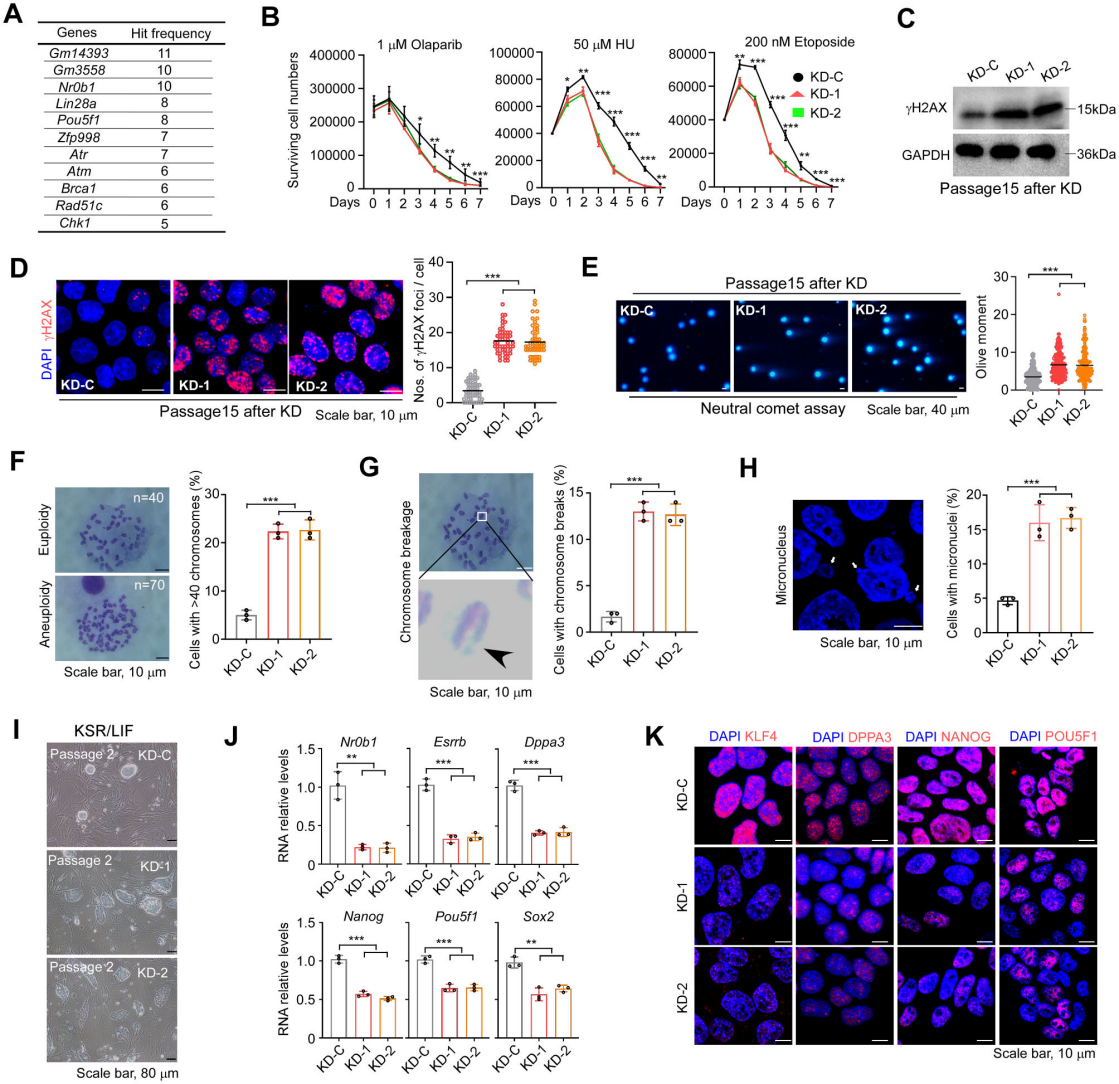
ZFP998 maintains naïve pluripotency and genomic stability in mESCs. (**A**) Top candidate genes and positive control genes identified from two independent CRISPR–Cas9 screens. The library targeted 579 mESC-specific genes. MAGeCK analysis identified genes whose knockout impaired cell fitness under mild DNA damage treatment. Known DDR genes (e.g. *Atm, Atr, Brca1, Chk1*) served as positive controls. *Zfp998* was among the top hits. (**B**) Cell viability assay for *Zfp998* knockdown control (KD-C) and two independent knockdown lines (KD-1, KD-2) mESCs treated with the indicated DNA-damaging agents: olaparib (a PARP inhibitor, 1 µM), hydroxyurea (HU, a nucleotide reductase inhibitor that inhibits DNA synthesis, 50 µM), or etoposide (a topoisomerase II inhibitor, 200 nM). Viability was assessed by direct cell counting using an automated cell counter. (**C**) Immunoblot analysis of γH2AX protein levels in KD-C and *Zfp998* KD mESCs. GAPDH served as a loading control. (**D**) Representative immunofluorescence images and quantification of γH2AX foci in KD-C and *Zfp998* KD mESCs. Nuclei were counterstained with DAPI. The graph shows the mean number of γH2AX foci per nucleus from at least 200 cells per condition across 30 fields of view. (**E**) Neutral comet assay showing increased DNA DSBs in *Zfp998* KD mESCs. The tail moment was quantified from at least 100 cells per group. (**F**) Chromosome number analysis in *Zfp998* KD and KD-C cells. Left panel, representative images of aneuploid metaphases of *Zfp998* KD and KD-C cells. Right panel, frequency of aneuploid metaphases. (**G**) Chromosome break analysis in *Zfp998* KD and KD-C cells. Left panel, representative images of chromosome breaks of *Zfp998* KD and KD-C cells. Right panel, frequency of chromosomal breaks. For panels (**F**) and (**G**), at least 50 metaphase spreads were analyzed per replicate across three independent experiments. (**H**) Micronuclei formation analysis. Left panel, representative images of micronuclei. Right panel, frequency of micronuclei. At least 50 visual fields containing 1000 cells were scored per replicate across three experiments. (**I**) Representative images showing the flattened colony morphology of *Zfp998* KD mESCs compared with the compact, dome-shaped colonies of KD-C cells in KSR/LIF medium. (**J**) Quantitative RT-PCR analysis of naïve (e.g. *Nrob1, Esrrb*, and *Dppa3*) and core (e.g. *Nanog, Pou5f1, Sox2*) pluripotency marker gene expression in KD-C and *Zfp998* KD mESCs. Expression levels were normalized to *Actb*. KD-C was set as 1. (**K**) Representative immunofluorescence images for the naïve (KLF4 and DPPA3) and core (NANOG and POU5F1) pluripotency markers in KD-C and *Zfp998* KD mESCs. Nuclei were counterstained with DAPI. All experiments in panels (**B**–**K**) were repeated at least three times with independent biological samples. Data are presented as mean ± SEM and analyzed using two-tailed Student’s *t-*test. *P < 0.05, **P < 0.01, ***P < 0.001.

In our initial attempt to identify regulators of genomic stability in mESCs, we conducted a customized CRISPR/Cas9-based loss-of-function screen for 579 genes that are upregulated in mESCs compared with those in other mouse organs (fold change >2) (Supplementary Data S3). ESCs were treated with various drugs, Etoposide, Hydroxyurea, Melphalan, Olaparib, 6-mercaptopurine, Topotecan, Zeocin, to induce DNA damage [23]. Genomic DNA was extracted from cells before and after drug selection, and the integrated sgRNA sequences were amplified by PCR and subjected to next-generation sequencing to quantify sgRNA abundance. Enrichment or depletion of sgRNAs under each condition was used to identify genes whose loss confers altered resistance to DNA damage (Supplementary Fig. S1A). A list of genes with well-known functions in DDR (e.g. *Atm, Atr, Brca1, Rad51c*, and *Chk1*) was included as positive controls [24]. Among the top candidate regulators with the highest hitting scores (Fig. 1A), we selected the transcription factor ZFP998 for downstream validation and investigation. This choice was based on two considerations: (i) transcription factors can play a determinant role in many cellular events by simultaneously regulating the expression of a wide range of target genes; and (ii) the function of ZFP998 remains largely unknown, except for a previous study reporting its involvement in suppressing mouse non-ecotropic endogenous retrovirus [25].

ZFP998 is a KRAB-containing zinc finger (KRAB-ZNF) protein. Quantitative RT-PCR showed that *Zfp998* expression was high in mESCs compared with that in other somatic tissues (Supplementary Fig. S1B). Moreover, *Zfp998* transcripts were most abundant in ICM of early embryos at embryonic day 3.5 (E3.5) [26] (Supplementary Fig. S1C). Although two isoforms are transcribed from *Zfp998* (Supplementary Fig. S1D), the long isoform is predominantly expressed in mESCs (Supplementary Fig. S1E). The long isoform is 2810 base pairs (bp) in length and encodes a full-length protein containing a KRAB domain and a Zinc finger domain, whereas the short isoform is 515 bp in length and produces a truncated protein containing only the KRAB domain (Supplementary Fig. S1D).

We knocked down (KD) the long isoform of *Zfp998* using two independent shRNAs in mESCs (C57BL/6J background) (Supplementary Fig. S1F). Consistent with the screening result, *Zfp998* KD mESCs showed decreased resistance to DNA-damaging drugs including olaparib (a PARP inhibitor), hydroxyurea (HU, a nucleotide reductase inhibitor that inhibits DNA synthesis) and etoposide (a topoisomerase II inhibitor) compared with the KD control (KD-C) (Fig. 1B), suggesting that ZFP998 is a regulator of DDR in mESCs. Concordantly, even under normal culture conditions, *Zfp998* KD mESCs failed to resolve endogenous DNA damage and contained elevated levels of DNA DSBs (Fig. 1C–E), aneuploidy (Fig. 1F), chromosome breakage (Fig. 1G), and micronuclei (Fig. 1H). Although basal γH2AX levels in mESCs vary across studies [27–30] due to methodological differences, we observed low baseline signals under our conditions (Fig. 1D, consistent with [30]), and importantly, *Zfp998* KD reproducibly increased γH2AX foci relative to paired controls.

Intriguingly, *Zfp998* KD ESC colonies at early passage (passage 2 or 3), regardless of whether they were cultured in conventional KSR/LIF medium or 2i/LIF condition, lost the typical dome-like shape and displayed a flattened morphology (Fig. 1I and Supplementary Fig. S1G). Notably, *Zfp998* KD ESCs could not survive in 2i/LIF condition and died within three passages (Supplementary Fig. S1G and H). KD ESCs maintained in KSR/LIF medium were able to survive. They displayed a reduced proliferation rate (Supplementary Fig. S1I) but remained positive for alkaline phosphatase (AP) staining (Supplementary Fig. S1J), indicating that they had exited the naïve state while retaining a certain level of pluripotency. Consistent with the colony morphological change, *Zfp998* KD ESCs cultured in KSR/LIF medium exhibited a significant decrease in the expression of naïve pluripotency genes (e.g. *Nr0b1, Esrrb, Dppa3, Nanog*, and *Klf4*) and a moderate reduction in core pluripotency genes (e.g. *Pou5f1, Sox2*) (Fig. 1J and K). Thus, ZFP998 is essential for naïve pluripotency and genomic stability in mESCs.

To validate the results obtained in *Zfp998* KD ESCs, we generated *Zfp998* KO mESCs (C57BL/6J background) via CRISPR/Cas9 (Supplementary Fig. S2A–C) and repeated the above experiments. Consistently, *Zfp998* KO recapitulated all the KD phenotypes, including increased sensitivity to DNA-damaging drugs (Supplementary Fig. S2D), accumulation of endogenous DNA DSBs and genomic instability (Supplementary Fig. S2E–J), loss of typical dome-shaped morphology (Supplementary Fig. S2K and L), cell death under 2i/LIF conditions (Supplementary Fig. S2L and M), reduced proliferation rate in KSR/LIF medium (Supplementary Fig. S2N), and decreased expression of pluripotency genes despite maintaining AP staining (Supplementary Fig. S2O–Q). To exclude the influence of genetic background, we also KD *Zfp998* in the R1 mESC line derived from 129 strain (Supplementary Fig. S3A). Similar phenotypes were observed (Supplementary Fig. S3B–F), demonstrating that the function of ZFP998 is independent of genetic background.

Mouse EpiSCs displayed a flattened morphology (Supplementary Fig. S2K) and barely expressed *Zfp998* (Supplementary Fig. S4A). Compared to EpiSCs, *Zfp998* KO cells exhibited more severe genomic instability (Supplementary Fig. S2E–J), but expressed relatively higher levels of pluripotency markers (Supplementary Fig. S2P and Q). To characterize the molecular differences between *Zfp998* KO ESCs and EpiSCs, we conducted RNA-seq. Principal components analysis (PCA) showed that WT, KO, and EpiSCs were clearly segregated, indicating a substantial difference in global gene expression profiles between *Zfp998* KO cells and EpiSCs (Supplementary Fig. S4B). Indeed, a comparison between KO and EpiSCs identified 1584 genes (corresponding to 1635 transcripts) and 729 genes (corresponding to 743 transcripts) that were up- and down-regulated in *Zfp998* KO ESCs, respectively (Supplementary Fig. S4C) (fold change >2, *q* < 0.05). Focusing on pluripotency and DDR genes, 38 stem cell maintenance genes were differentially expressed (Supplementary Data S4), the majority of which displayed higher expression in KO than in EpiSCs (Supplementary Fig. S4D). Additionally, 92 differentially expressed DDR genes were identified (Supplementary Data S4). Although most of them showed higher expression in *Zfp998* KO ESCs (Supplementary Fig. S4E), the expression of some key DNA damage repair genes (e.g. *Smc3, Ddb2, Rad51b*, and *Ube2t*) was lower in KO cells. This may account for the severe genomic instability phenotype observed in KO cells.

### ZFP998 regulates the expression of a wide range of genes involved in naïve pluripotency and genomic stability maintenance

**Figure 2.**
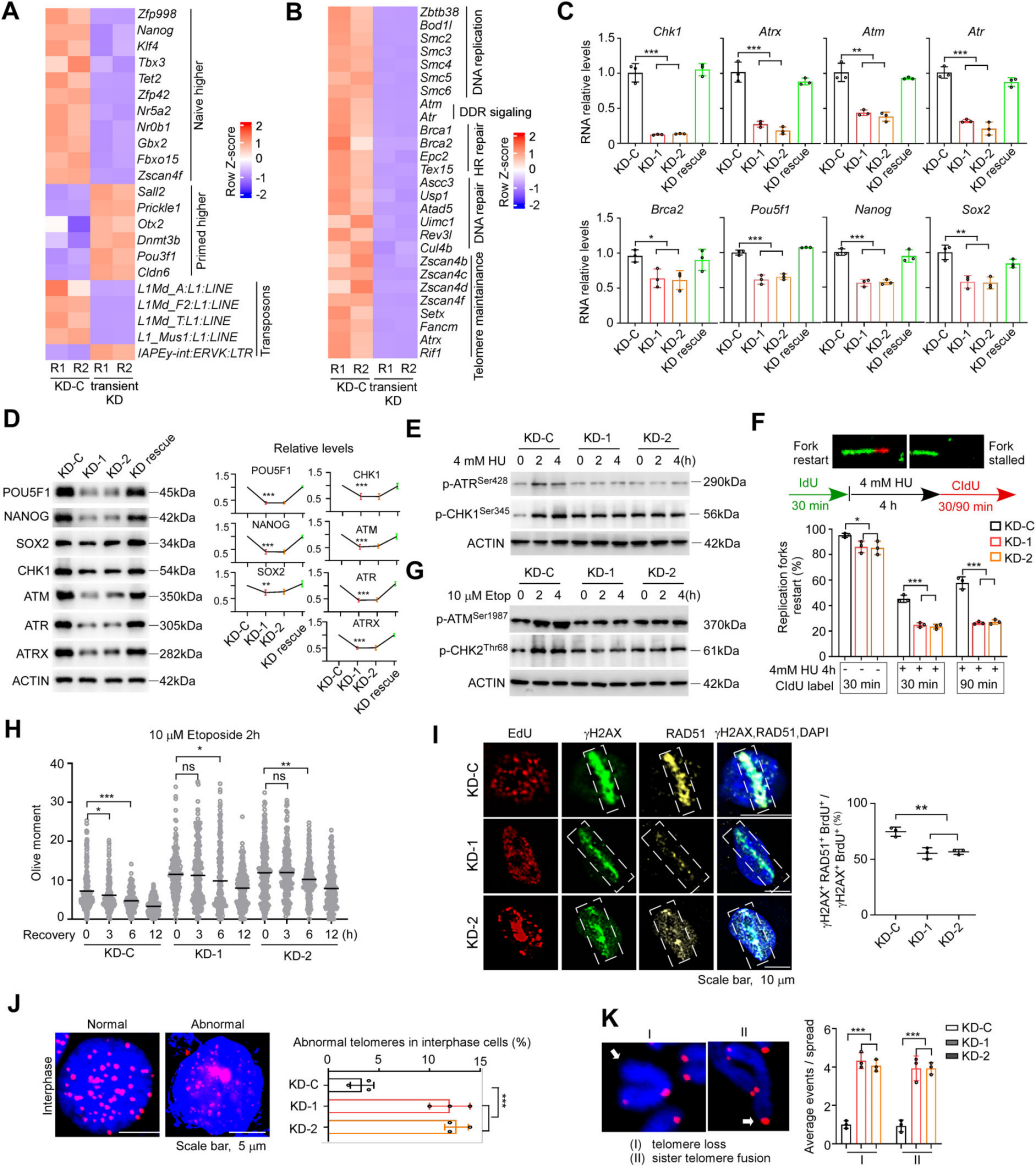
ZFP998 regulates the expression of a wide range of genes involved in naïve pluripotency and genome stability maintenance. Heatmaps depicting the expression changes of representative (**A**) naïve pluripotency-associated genes and (**B**) DDR genes in control (KD-C) and transient *Zfp998* KD mESCs (3 days post-shRNA transfection). (**C**) Quantitative RT-PCR validation of key DDR (e.g. *Chk1, Atr, Atm, Atrx*, and *Brca2*) and pluripotency (e.g. *Pou5f1, Sox2, Nanog*) gene expression changes in KD-C, *Zfp998* KD, and KD mESCs rescued by re-expression of FLAG-ZFP998 (KD rescue). mRNA levels were normalized to *Actb*. KD-C was set as 1. (**D**) Immunoblot analysis validating the protein expression changes of key DDR (e.g. CHK1, ATR, ATM, and ATRX) and pluripotency (e.g. POU5F1, SOX2, NANOG) factors in the indicated cell lines. *ACTIN* served as the loading control for quantification. (**E**) Immunoblot analysis of the ATR-CHK1 signaling pathway in KD-C and *Zfp998* KD mESCs, showing compromised pathway activation upon KD. (**F**) DNA fiber assay analysis of replication fork dynamics. Representative images of DNA fibers are shown (top panel). Quantification revealed impaired stalled fork restart in *Zfp998* KD mESCs compared with KD-C. Data are from at least 200 fibers analyzed across three independent experiments. (**G**) Immunoblot analysis of the ATM-CHK2 signaling pathway in KD-C and *Zfp998* KD mESCs, showing compromised pathway activation. (**H**) Neutral comet assay assessing the kinetics of DNA DSB repair. Cells were treated with etoposide (10 µM) for 2 h and allowed to recover for the indicated times. Repair was delayed in *Zfp998* KD mESCs. Tail moment was quantified from at least 100 cells per condition. (**I**) Laser micro-irradiation assay to measure HR repair efficiency. HR activity is indicated by the co-localization of RAD51 foci with γH2AX tracks in EdU-positive (S-phase) cells. The left panel shows the representative images of micro-irradiation assay. Quantification is shown on the right. At least 50 cells were analyzed per group. Telomere fluorescence *in situ* hybridization showing increased telomere abnormalities in *Zfp998* KD mESCs at (**J**) interphase and (**K**) metaphase. Representative images are shown alongside quantification. For panel (J), at least 50 interphase cells were scored; for panel (**K**), at least 50 metaphase spreads were analyzed per group. All experiments shown in panels (**C**–**K**) were repeated at least three times with independent biological samples. Data are presented as mean ± SEM and analyzed using two-tailed Student’s *t-*test. *P < 0.05, **P < 0.01, and ***P < 0.001.

To further understand how ZFP998 regulates pluripotency and genomic stability in mESCs, we examined the differential gene expression between transient *Zfp998* KD (3 days post shRNA transfection) and KD-C cells by RNA-seq. A total of 3698 DEGs (fold change >2, *q* < 0.05) were detected between KD-C and transient *Zfp998* KD ESCs, with 2203 genes down-regulated and 1499 genes up-regulated in transient KD cells, respectively (Supplementary Fig. S5A and Supplementary Data S5). DEGs were also identified between KD-C and KO ESCs (Supplementary Fig. S5B and Supplementary Data S6). The down-regulated genes in *Zfp998-*deficient cells (transient KD or KO) were consistently enriched for the processes related to genomic stability regulation and stem cell maintenance, whereas the up-regulated genes played roles in processes including differentiation and organism development (Supplementary Fig. S5A and B). Specifically, a panel of typical naïve pluripotency genes including protein-coding genes and transposable elements was down-regulated in transient KD and KO mESCs, whereas primed pluripotency markers displayed up-regulation (Fig. 2A and Supplementary Fig. S5C). Importantly, many DDR genes from AmiGO (http://amigo.geneontology.org/amigo) were decreased in transient *Zfp998* KD (160/805) or KO (138/805) cells (Supplementary Data S5 and S6). The affected DDR genes fell into the categories of DNA damage signaling (e.g. *Atm, Atr*), DNA replication and repair, homologous recombination (HR)-mediated repair pathway, telomere maintenance, and apoptosis (Fig. 2B and Supplementary Fig. S5D). The expression changes of a subset of key DDR genes and pluripotency genes were validated by quantitative RT-PCR (Fig. 2C) and immunoblotting (Fig. 2D). To exclude possible off-target effects, we re-introduced ZFP998 into KD ESCs and established a rescue cell line (KD rescue) (Supplementary Fig. S5E). Notably, KD rescue cells recovered the dome-shaped morphology (Supplementary Fig. S5F), and reversed the whole-genome gene expression changes (Supplementary Fig. S5G). The expression recovery of some DDR and pluripotency genes was validated (Fig. 2C and D). Thus, these defects were *bona fide* induced by *Zfp998* depletion.

The impaired expression of a wide range of DDR genes in *Zfp998-*deficient (KD or KO) ESCs suggests that ZFP998 safeguards genomic stability via multiple pathways. DNA replication stress is a major source of endogenous DNA damage. ATR-CHK1 signaling pathway plays a central role in coordinating replication stress responses [31], by arresting cell cycle as well as protecting and repairing the stalled forks. The activation of this pathway, monitored by the phosphorylation of CHK1 at Ser345 [32], was compromised in *Zfp998* KD or KO ESCs (Fig. 2E and Supplementary Fig. S6A). Concordantly, the S-phase checkpoint activation was inefficient (Supplementary Fig. S6B), and stalled fork restart was drastically decreased as measured by DNA fiber assay [33] (Fig. 2F and Supplementary Fig. S6C). ATM-CHK2 signaling pathway is critical for sensing and repairing DNA DSBs [34]. In *Zfp998-*deficient ESCs, the activation of this pathway (Fig. 2G and Supplementary Fig. S6D) and the repair of DNA DSBs were compromised (Fig. 2H and Supplementary Fig. S6E). In particular, to assess high-fidelity HR-mediated DSB repair, we measured the recruitment of recombinase RAD51 to DSB sites marked by γH2AX in S-phase ESCs following laser micro-irradiation. In *Zfp998* KD or KO ESCs, the HR repair efficiency was significantly decreased (Fig. 2I and Supplementary Fig. S6F). Genes including *Rif1, Zscan4, Setx*, and *Atrx* are essential for telomere integrity maintenance [35–38]. Loss of telomere integrity can induce telomere abnormalities. Indeed, higher rates of telomere abnormality were detected at interphase and metaphase in *Zfp998* KD (Fig. 2J and K) or KO cells (Supplementary Fig. S6G and H). Of note, consistent with the observation that *Zfp998* KO cells exhibited more severe genomic instability than EpiSCs (Supplementary Fig. S2E–J), KO cells had lower capacities of DDR and repair than EpiSCs (Supplementary Fig. S6). Taken together, these lines of evidence support the dual roles of ZFP998 in regulating naïve pluripotency and genomic stability in mESCs.

### ZFP998 binds to promoters/enhancers of a subset of pluripotency and DDR genes and modulates their activities

**Figure 3.**
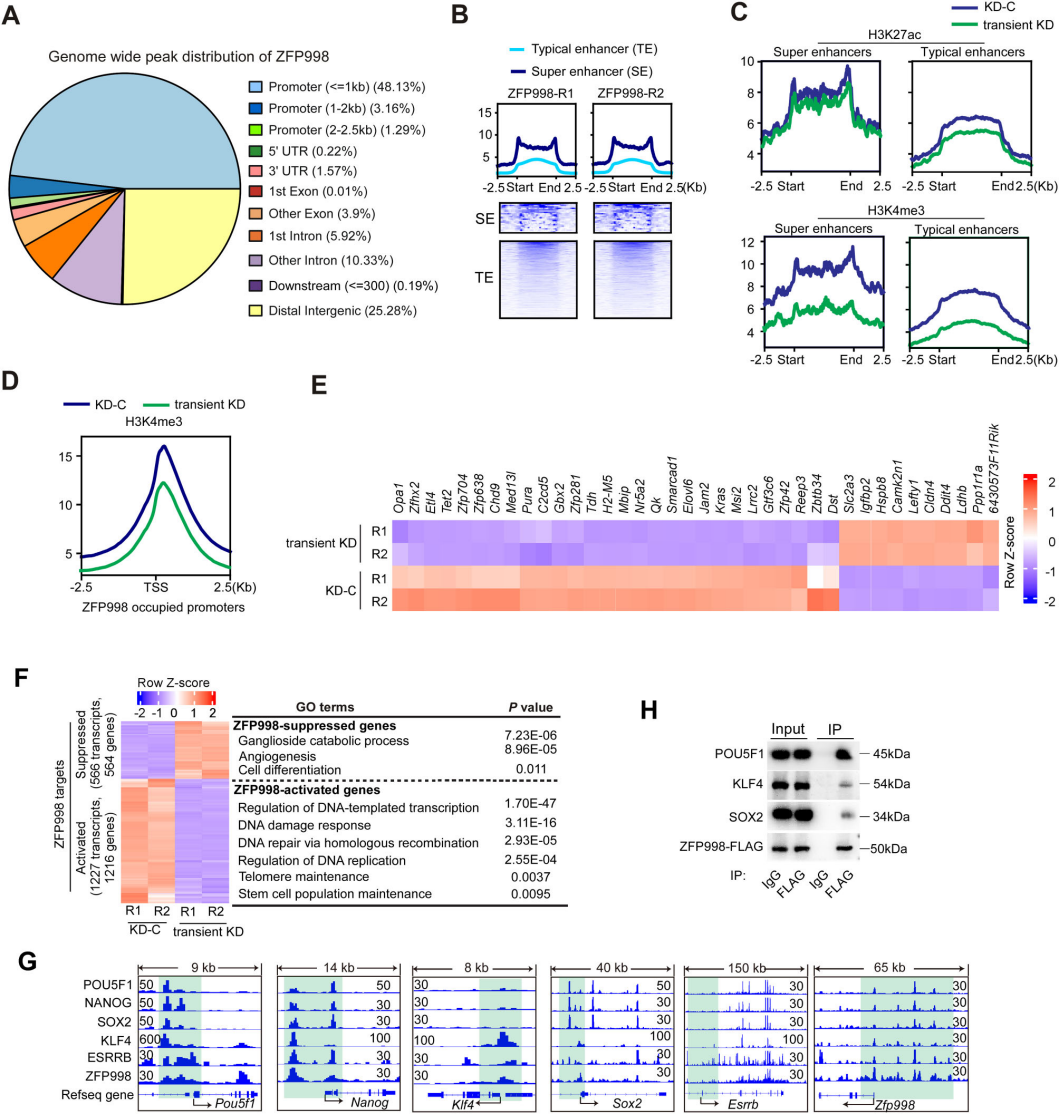
ZFP998 binds to promoters/enhancers to regulate the expression of pluripotency and DDR genes. (**A**) Distribution of genome-wide ZFP998 binding peaks across different genomic regions (promoters, exons, introns, and intergenic regions) in mESCs, as determined by CUT&Tag. Data are presented as a percentage of total peaks. CUT&Tag was performed with an anti-FLAG antibody in FLAG-ZFP998-expressing mESCs. (**B**) ZFP998 binding peak intensities at typical enhancers and super-enhancers in mESCs. Data were from two replicates (R1 and R2). (**C**) Changes in H3K27ac and H3K4me3 peak intensities at typical enhancers and super-enhancers following transient *Zfp998* KD. Signal was normalized using RPKM. (**D**) Transient *Zfp998* KD decreased the peak intensity of H3K4me3 at ZFP998-occupied promoter regions. Promoters were defined as regions ±2.5 kb from TSS. (**E**) Heatmap of mESC identity gene expression changes upon transient *Zfp998* KD. (**F**) Heatmap of ZFP998 target gene expression in KD-C versus transient *Zfp998* KD mESCs. ZFP998 upregulated and downregulated target genes are shown separately with corresponding GO enrichment terms. (**G**) Integrative Genomics Viewer (IGV) browser tracks showing the co-occupancy of POU5F1, NANOG, SOX2, KLF4, ESRRB, and ZFP998 at each other’s promoter regions. ChIP-seq datasets of POU5F1, NANOG, SOX2, ESRRB, and KLF4 were retrieved from the GEO database under the accession numbers GSM1082340, GSM1082342, GSM1082341, GSM288355, as well as GSM6597312 and GSM6597313, respectively [39, 40, 41, 42]. (**H**) Co-immunoprecipitation (co-IP) combined with immunoblotting validated the physical interaction between ZFP998 and the core pluripotency factors POU5F1, SOX2, and KLF4. The experiments were performed with at least three biological replicates. Data in panels (**A**–**F**) are derived from two independent biological replicates.

We moved on to identify the binding regions of ZFP998 on chromatin using mESCs expressing FLAG-tagged ZFP998 (Supplementary Fig. S5E). For CUT&Tag experiments, we included two controls: one using the anti-FLAG antibody in isogenic WT cells, and another using IgG in FLAG-tagged ZFP998-expressing cells. Both control CUT&Tag experiments yielded extremely low background signal with minimal chromatin enrichment (Supplementary Fig. S7A). Therefore, in the following analyses, we utilized IgG in FLAG-tagged ZFP998-expressing cells as the control to correct for background. CUT&Tag analysis obtained highly reproducible data between two biological replicates (Supplementary Fig. S7B). Among the 19 822 ZFP998 binding sites (Supplementary Data S7), ~53% were located within 2.5 kb of transcription start sites (TSS) (promoter regions), and ~25% were at distal intergenic regions where enhancers are often located (Fig. 3A). At promoter regions, 75.8% of ZFP998 binding sites resided at the TSS. To better characterize the binding regions of ZFP998, we examined the genome-wide distribution profiles of H3K4me3 and H3K27ac in KD-C and transient KD (3 days post shRNA transfection) ESCs. Integrative analyses revealed that 99.7% of ZFP998 binding regions were decorated by H3K4me3, suggesting that ZFP998 was predominantly associated with open chromatin. Typical enhancers and super-enhancers in mESCs have been identified in a previous study [39]. Of note, ZFP998 bound to 24.4% (2086/8563) of typical enhancers and 94.4% (218/231) of super-enhancers, and the binding intensity on super-enhancers was higher than that on typical enhancers (Fig. 3B). Thus, ZFP998 occupies many regulatory elements including promoters, typical enhancers, and super-enhancers.

Further, we examined whether ZFP998 regulated the activity of associated enhancers and promoters by comparing the peak intensities of H3K4me3 and H3K27ac between KD-C and transient KD ESCs. Notably, the peak intensities at ZFP998-associated typical enhancers and super-enhancers were reduced in transient *Zfp998* KD ESCs (Fig. 3C). Similarly, the H3K4me3 intensity at ZFP998-occupied promoters was lower in transient KD ESCs than in KD-C cells (Fig. 3D). These results indicated that ZFP998 bound to promoters, typical enhancers, and super-enhancers and regulated their activity.

Super-enhancers drive the expression of cell identity genes. In mESCs, 231 super-enhancers control the expression of 210 ESC key identity genes [39]. Among these identity genes, 38 genes exhibited expression changes upon transient *Zfp998* KD (fold change >2) (Fig. 3E and Supplementary Data S8), indicating that ZFP998 directly regulated the expression of these identity genes. As a control, transient KD of *Pou5f1* (Supplementary Fig. S7C), a master transcription factor that binds to super-enhancers, affected the expression of 48 mESC key identity genes (Supplementary Fig. S7D). To identify additional target genes whose expression is subject to the direct regulation by ZFP998, we focused on ZFP998-associated promoter regions given that enhancer-modulated genes are difficult to predict. ZFP998-occupied promoters were adjacent to 9154 genes (ZFP998-associated genes) (Supplementary Data S7). Integrative analyses of the RNA-seq and CUT&Tag data revealed that 1793 transcripts from 1779 genes showed significant expression changes (fold change >2, *q* < 0.05) upon transient *Zfp998* KD. These genes were then referred to as ZFP998 target genes (Supplementary Data S9). ZFP998-activated genes (decreased in transient KD ESCs) play roles in the processes of transcription regulation (e.g. *Foxd3, Foxp1*), DDR (e.g. *Atm, Chk1*, and *Fancm*), DNA replication (e.g. *Orc3, Smarca5*, and *Smc3*), HR-mediated DNA DSB repair (e.g. *Brca1, Rnf138, Ino80, Epc1, Gen1*, and *Mbtd1*), telomere maintenance (e.g. *Rif1, Rad50, Terf1, Xrn1*, and *Zfp827*), and stem cell maintenance (e.g. *Rif1, Bmpr1a, Apc, Zcchc11*, and *Cdc73*) (Fig. 3F and Supplementary Fig. S7E and F). On the other hand, target genes repressed by ZFP998 were involved in processes such as organismal development (e.g. *Bmp6, Fgfr4, Snai1*, and *Pdgfb*), carbohydrate metabolism (e.g. *Hexa, Hexb, Pdk2*, and *Pgm2*) and aging (e.g. *Tspo, Apaf1*, and *Cd68*) (Fig. 3F and Supplementary Fig. S7G and H).

The observations that ZFP998 bound to super-enhancers and regulated ESC key identity gene expression suggested that ZFP998 is a novel component of the core naïve pluripotency regulatory loop in mESCs. The well-known pluripotency auto-regulatory loop is composed of master regulators POU5F1, SOX2, NANOG, KLF4, and ESRRB [43], which, together with mediators, establish the super-enhancers [39]. These master transcription factors have been shown to form an interconnected loop in which they bind as a group to the promoter/enhancer of each gene to form regulatory loops [39]. Indeed, the promoter/enhancer regions of *Pou5f1, Klf4, Sox2, Nanog*, and *Esrrb* displayed strong ZFP998 binding peaks (Fig. 3G). By analyzing the archived data [39], we also detected binding peaks of POU5F1, SOX2, NANOG, KLF4, and ESRRB at the promoter region of *Zfp998* (Fig. 3G). Moreover, immunoprecipitation analysis revealed that ZFP998 physically associated with POU5F1, KLF4, and SOX2 (Fig. 3H). Taken together, these lines of evidence support that ZFP998 is a novel component of the core pluripotency regulatory network. By simultaneously regulating many DDR genes, ZFP998 orchestrates both naïve pluripotency and genomic stability in mESCs.

### ZFP998 interacts with TRIM28, DDX5, and P300 at regulatory regions

**Figure 4.**
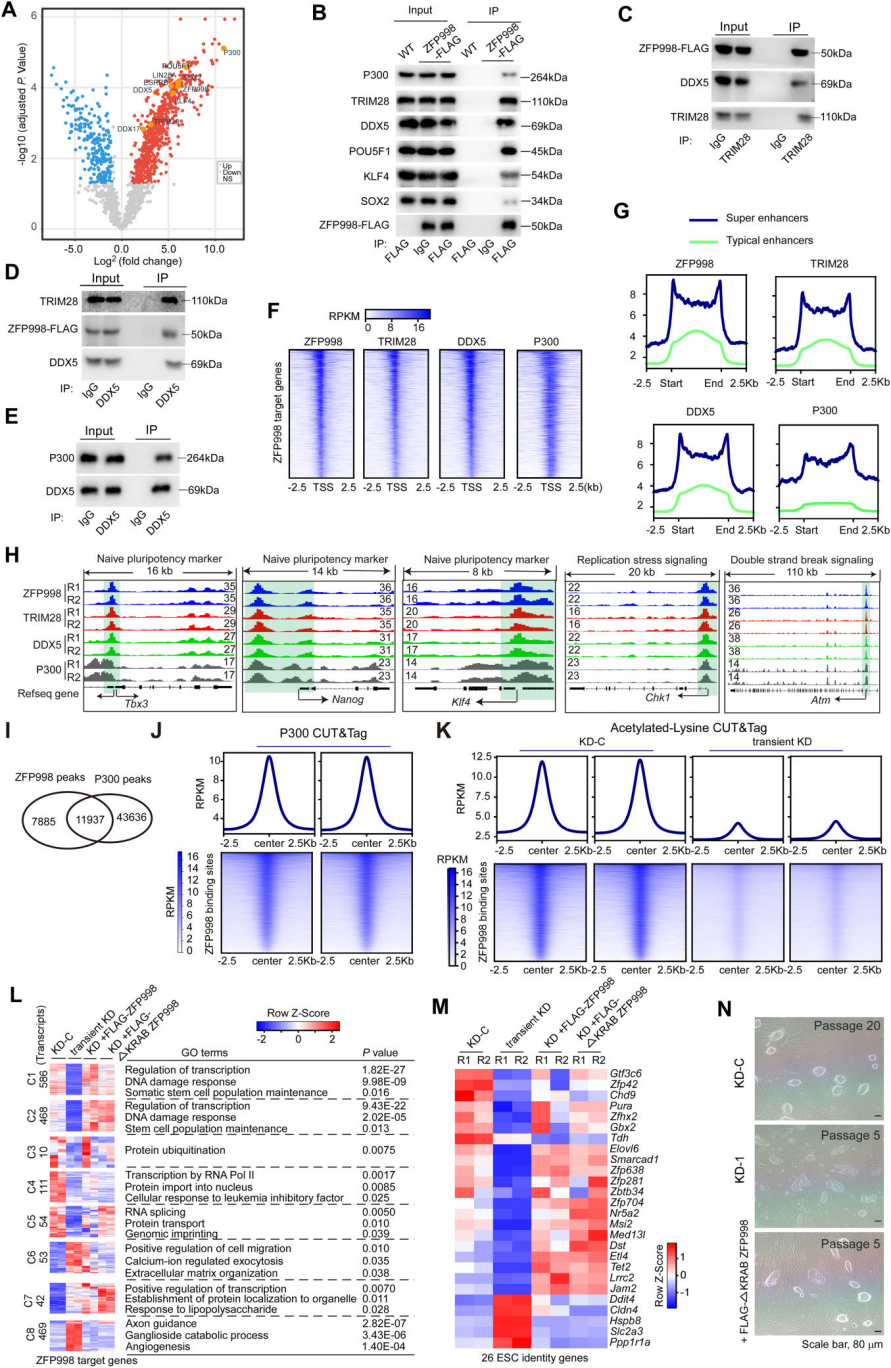
ZFP998 interacts with TRIM28, DDX5 and P300 at regulatory regions. (**A**) Volcano plot analysis of ZFP998 interacting proteins identified by co-immunoprecipitation followed by MS. Co-immunoprecipitation was performed in FLAG-ZFP998-expressing mESCs and WT mESCs using an anti-FLAG antibody. Differentially enriched proteins were identified using limma (adjusted *P-*value < 0.05 and fold change >2 were considered statistically significant). Red dots indicate proteins enriched in FLAG-ZFP998-expressing mESCs. Blue dots indicate proteins enriched in WT mESCs. Samples are from three independent biological replicates. (**B**) Validation of the interaction between FLAG-ZFP998 and P300, TRIM28, POU5F1, DDX5, KLF4, or SOX2 by co-immunoprecipitation (co-IP) using a FLAG antibody. (**C**) Validation of the interaction between TRIM28 and ZFP998 or DDX5 by co-IP using a TRIM28 antibody. (**D**) Validation of the interaction between DDX5 and ZFP998 or TRIM28 by co-IP using a DDX5 antibody. (**E**) Validation of the interaction between DDX5 and P300 by co-IP using a DDX5 antibody. (**F**) Heatmaps show the binding intensity of ZFP998, DDX5, TRIM28, and P300 around ZFP998 target genes are presented. (**G**) Enrichment of ZFP998, TRIM28, DDX5, and P300-binding site around typical enhancers and super-enhancers in mESCs is shown. (**H**) IGV browser tracks show co-localization of ZFP998, TRIM28, DDX5, and P300 at promoters of key naïve pluripotency genes (*Tbx3, Nanog* and *Klf4*) and DDR genes (*Chk1* and *Atm*). (**I**) Venn diagram shows that 60% of ZFP998 binding sites overlap with P300 sites. (**J**) CUT&Tag heatmaps showing P300 enrichment around ZFP998 binding sites. Genomic regions are centered on ZFP998 peaks. Two biological replicates are shown for each condition (left and right columns within each heatmap). Signals are normalized as RPKM. (**K**) CUT&Tag heatmaps showing acetylated lysine signal intensities around ZFP998 binding sites. Acetylated lysine intensities at ZFP998 target binding sites were decreased following transient *Zfp998* KD. Two biological replicates are shown per condition. Signals are normalized as RPKM. Rescue of gene expression in *Zfp998* KD mESCs by re-expression of WT ZFP998 or the ΔKRAB mutant ZFP998. Heatmaps show expression changes of ZFP998 target genes (**L**) and mESC identity genes (**M**). Most genes show similar rescue by both constructs. (**N**) Representative images of colony morphology rescue. The ΔKRAB mutant restored dome-like morphology in the majority of colonies. Experiments in panels (**F**–**M**) were performed with two independent biological replicates. Experiments were repeated at least three times from independent biological samples in panels (**A**–**E**) and (**N**).

To understand how ZFP998 regulates the enhancer/promoter activity and the expression of associated target genes, we searched for its interacting proteins by co-immunoprecipitation combined with MS analysis. A list of interaction partners including TRIM28, RNA-binding proteins (RBPs) DDX5 and DDX17, the histone acetyltransferase CBP/p300 (P300), and the core pluripotency factors, POU5F1, SOX2, ESRRB and KLF4, was identified (Fig. 4A and Supplementary Data S10). Of interest, DDX5 (also known as p68) and DDX17 are able to regulate transcription by associating with key components of the chromatin remodeling and transcriptional machinery, such as P300, histone deacetylases and RNA polymerase II (Pol II) [44–46]. TRIM28 can either activate or suppress gene expression depending on the context [47–49]. The physical association of ZFP998 with these proteins was validated by co-immunoprecipitation followed by immunoblotting (Fig. 4B–D). The interaction of DDX5 with P300 was also verified (Fig. 4E).

We then investigated whether ZFP998, TRIM28, DDX5, and P300 share overlapping binding regions on chromatin. CUT&Tag analysis of TRIM28, DDX5, and P300 (Supplementary Fig. S8A–C) revealed that over 45% of ZFP998 binding sites were co-occupied by TRIM28, DDX5, and P300 (Supplementary Fig. S8D), further supporting their functional co-localization at chromatin. Specifically, 70% of ZFP998-associated genes (6218/9154) (Supplementary F ig. S8E) and 72% of ZFP998 target genes (1280/1779) (Fig. 4F) contained binding peaks of TRIM28, DDX5, and P300 at their promoter regions. Moreover, a large proportion of ZFP998-targeted typical enhancers (43.3%, 903/2086) and super-enhancers (77.5%, 169/218) were co-occupied by TRIM28 and DDX5, although only a small fraction of ZFP998-targeted typical enhancers (8.6%, 181/2086) and super-enhancers (16.5%, 36/218) were co-occupied by TRIM28, DDX5, and P300 (Fig. 4G). Thus, ZFP998 interacts with TRIM28, DDX5, and P300 at shared promoter/enhancer regions. The co-occupancy of ZFP998, TRIM28, DDX5, and P300 on the promoters of several naïve pluripotency and DDR genes is shown (Fig. 4H and Supplementary Fig. S8F). The core function of P300 is to catalyze the acetylation of various histone lysine residues, thereby driving gene activation [50]. We thus examined the influence of ZFP998 depletion on histone lysine acetylation. Focusing on the overlapping binding sites of ZFP998 and P300 (Fig. 4I and J, and Supplementary Fig. S8G), CUT&Tag analysis of acetylated lysines revealed that loss of *Zfp998* resulted in decreased acetylation levels at these co-occupied sites (Fig. 4K and Supplementary Fig. S8G and H), further supporting that ZFP998 recruits P300 to its target regions to regulate epigenetic modifications and gene expression.

We also performed motif enrichment analysis for ZFP998 binding sites. *De novo* motif analysis identified a novel binding motif for ZFP998 (Supplementary Fig. S8I). Moreover, the binding motifs for several transcription factors including SMAD4, ZFX, and KLF4, were also identified (Supplementary Fig. S8I). SMAD4 is a core component of the BMP signaling pathway [51]. Both BMP signaling and ZFX are key regulators of mESC self-renewal [52, 53]. These observations suggest that ZFP998 might collaborate with these key transcription factors to carry out its functions.

### TRIM28 is dispensable for ZFP998 to regulate gene expression

TRIM28 either activates or suppresses gene expression depending on the distinct contexts. We then focused on TRIM28 to clarify its role in mediating the function of ZFP998. To this end, we generated a mutant ZFP998 lacking the TRIM28-interacting KRAB domain and expressed the FLAG-tagged mutant protein (ΔKRAB ZFP998) in *Zfp998* KD ESCs (Supplementary Fig. S9A). As a control, re-expression of full-length ZFP998 restored the dome-shaped colony morphology (Supplementary Fig. S5F), the mRNA and protein expression of selected DDR and pluripotency genes (Fig. 2C and D), and the global gene expression profile (Supplementary Fig. S5G). Intriguingly, although ΔKRAB ZFP998 failed to interact with TRIM28, it retained its association with DDX5 (Supplementary Fig. S9B), and displayed even stronger binding peaks on typical enhancers, super-enhancers, and promoter regions of ZFP998 target genes than full-length ZFP998 did (Supplementary Fig. S9C). Moreover, ΔKRAB ZFP998 had a similar capacity to full-length ZFP998 in rescuing the expression changes of most ZFP998 target genes (Fig. 4L and Supplementary Fig. S9D) and ZFP998-regulated ESC identity genes (Fig. 4M, Supplementary Fig. S9E, and Supplementary Data S11). Notably, re-expression of ΔKRAB ZFP998 restored the dome-like morphology in more than 50% of KD ESC colonies (Fig. 4N). These observations altogether suggest that TRIM28 is not essential for ZFP998 to regulate gene expression.

### ZFP998 is able to reset primed EpiSCs to naïve pluripotency state

**Figure 5.**
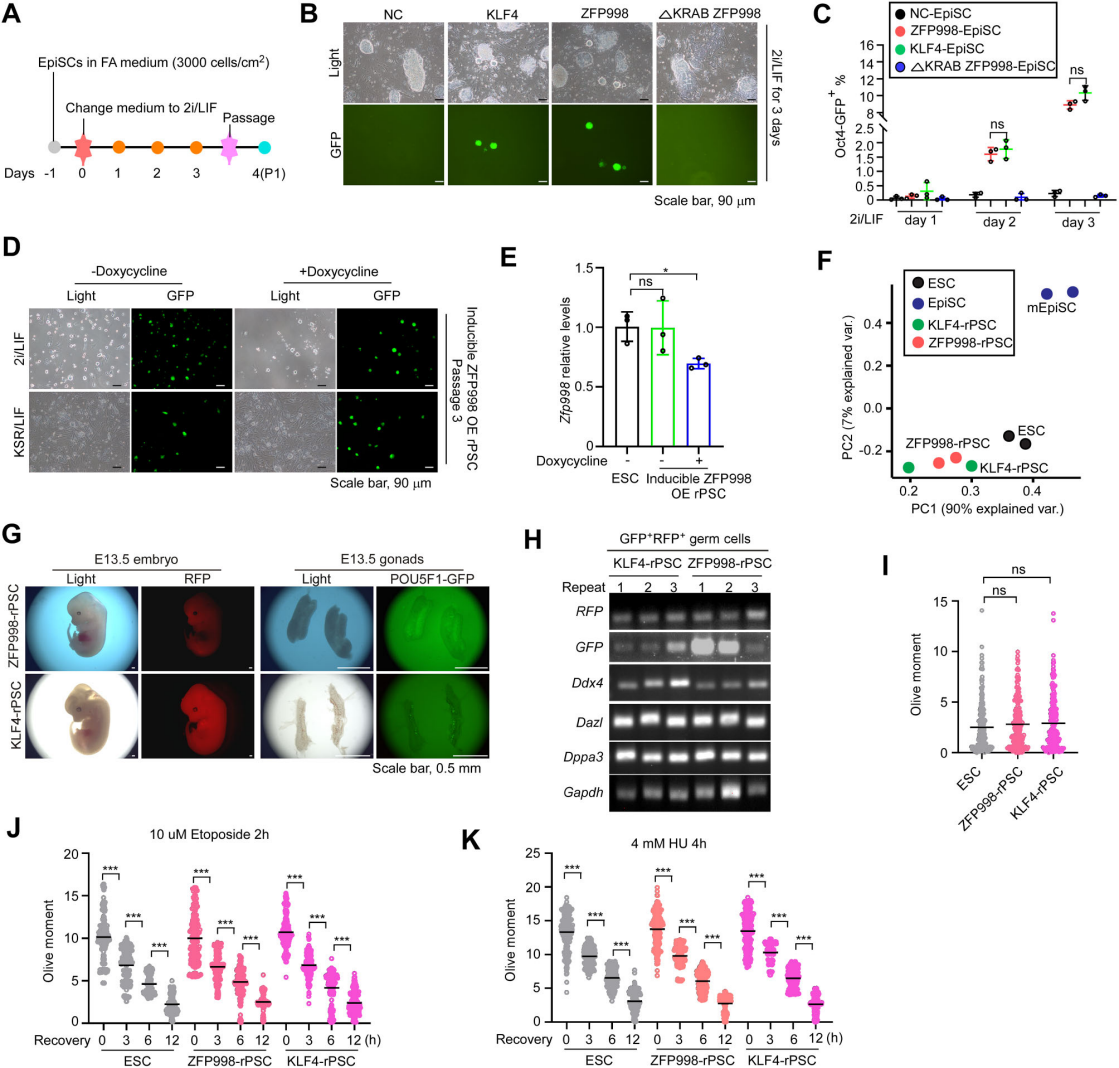
ZFP998 is able to reset primed EpiSCs to a naïve pluripotency state. (**A**) Schematic of the experimental workflow to reset mouse EpiSCs to naïve pluripotency. (**B**) Representative images on day 3 of reprogramming. Mouse EpiSCs were transfected with PiggyBac transposon vectors for the expression of KLF4, WT ZFP998, the ΔKRAB ZFP998 mutant, or an empty vector control (NC). Conversion to the naïve state was monitored using a *Pou5f1*-△PE-GFP reporter. GFP signal indicates activation of the naïve-state reporter. Images were acquired using identical exposure settings for all conditions. (**C**) Quantification of reprogramming efficiency. The percentage of GFP-positive (GFP^+^) cells was determined by flow cytometry (FACS) on days 1, 2, and 3. Data points represent biological replicates (*n* = 3). (**D**) Assessment of naïve state stability. Mouse EpiSCs were transfected with a PiggyBac transposon vector carrying a doxycycline-inducible ZFP998 expression cassette and then transferred to 2i/LIF medium to induce reprogramming to naïve pluripotency. The resulting ZFP998-induced rPSCs were subsequently passaged three times and maintained in either 2i/LIF or KSR/LIF medium in the absence (−) or presence (+) of doxycycline. Representative images show that the rPSCs retained the characteristic dome-shaped colony morphology and sustained GFP reporter expression, confirming stable maintenance of the naïve state. (**E**) Quantitative RT-PCR analysis of endogenous *Zfp998* mRNA expression in established rPSCs. RNA was harvested from cells cultured with or without doxycycline. Expression levels were normalized to *Actb* and are presented relative to mESCs (set to 1). Data are presented as mean ± SEM from three independent experiments. (**F**) PCA of global transcriptional profiles. RNA-seq was performed on parental EpiSCs, mESCs, and established rPSCs generated with KLF4 or ZFP998 (two biological replicates per group). Percentages indicate the variance explained by each principal component (PC). (**G**) *In vivo* chimera formation assay. Representative images of E13.5 embryos generated by eight-cell aggregation with KLF4-rPSCs or ZFP998-rPSCs (donor cells express constitutive RFP and *Pou5f1*-△PE-GFP germ cell reporter). Low-magnification view shows widespread chimerism (RFP, red). High-magnification view of the gonad (boxed area) shows GFP-positive donor-derived primordial germ cells (arrows). (**H**) RT-PCR analysis of germ cell marker expression. RNA was extracted from the gonad cells and expression of the indicated markers was analyzed by RT-PCR. A representative gel from three independent experiments is shown. (**I**) Assessment of baseline DNA damage. Endogenous DNA DSB levels in the indicated cell types were measured by the neutral comet assay. The tail moment was quantified from at least 100 cells per group across three independent experiments. DDR assays. Cells were treated with etoposide (10 µM for 2 h; **J**) or hydroxyurea (HU, 4 mM for 4 h; **K**) and allowed to recover for the indicated times. DSB repair kinetics were assessed by the neutral comet assay. The percentage of initial damage remaining was plotted over time. Data points represent mean ± SEM from three independent experiments. All data are presented as mean ± SEM and analyzed using two-tailed Student’s *t-*test. *P < 0.05, ***P < 0.001; ns, not significant. Experiments were repeated at least three times from independent biological samples.

The above lines of evidence indicate that ZFP998 simultaneously maintains naïve pluripotency and genomic stability in mESCs. It is involved in recruiting the histone acetyltransferases CBP/P300 to its binding sites, thereby promoting acetylation. These observations prompted us to further investigate whether ZFP998, like other transcription factors such as KLF4 [54], NR5A2 [55], and ESRRB [56], has the potential to drive the acquisition of naïve pluripotency and high genomic stability. To test this hypothesis, we utilized mouse EpiSCs derived from mouse embryos carrying a *Pou5f1*-GFP (OG2) reporter, i.e. a GFP reporter driven by the distal enhancer of *Pou5f1* that is indicative of naïve pluripotency [57]. EpiSCs do not express auxiliary pluripotency factors including ESRRB, KLF4, KLF2, and TBX3, and they are difficult to spontaneously convert to a naïve pluripotent state when cultured in ESC medium [54, 56, 58]. We expressed ZFP998 or ΔKRAB ZFP998 in EpiSCs, and included KLF4 expression as a positive control (Supplementary Fig. S10A–C). After transfer to 2i/LIF medium (Fig. 5A), cells expressing KLF4 (positive control) or ZFP998 formed GFP^+^ colonies on day 2, and the GFP signal became stronger on day 3 (Fig. 5B). Fluorescence-activated cell sorting (FACS) analysis revealed that forced expression of ZFP998 or KLF4 gradually and significantly increased the GFP^+^ cell population with similar efficiency (Fig. 5C). These GFP^+^ reprogrammed PSC colonies could be stably expanded and maintained in 2i/LIF or conventional KSR/LIF conditions (Supplementary Fig. S10D). To our surprise, ΔKRAB ZFP998 was unable to convert EpiSCs to a naïve pluripotent state, indicating that, unlike in the maintenance of naïve pluripotency, the KRAB domain was essential for the primed-to-naïve reprogramming (Fig. 5B and C).

To determine whether exogenous ZFP998 is required to maintain rPSCs, we established a cell line with doxycycline-inducible ZFP998 expression (Supplementary Fig. S10E and F) and repeated the experiments. Intriguingly, the removal of doxycycline after GFP^+^ colonies were established had no influence on the maintenance or expansion of these cells (Fig. 5D), indicating that ZFP998 was no longer required once the naïve state was established. This may be due to the successful induction of endogenous *Zfp998* expression (Fig. 5E).

We next evaluated the properties of these rPSCs. Like ESCs, both KLF4- and ZFP998- converted PSCs (named as KLF4-rPSCs and ZFP998-rPSCs, respectively) expressed typical naïve pluripotency markers (Supplementary Fig. S10G and H). KLF4-rPSCs and ZFP998-rPSCs were molecularly related to each other and exhibited partial ESC-like molecular features, rather than fully resembling ESCs (Fig. 5F). Specifically, the majority of pluripotency- and DDR-relevant genes exhibited similar expression levels across rPSCs and ESCs (Supplementary Fig. S10I and J, and Supplementary Data S12). To functionally validate the naïve pluripotency of ZFP998-rPSCs, we examined whether they could differentiate into germ cells in chimeric embryos. ZFP998-rPSCs or KLF4-rPSCs (control) expressing RFP (Supplementary Fig. S10K) were microinjected into 8-cell embryos. Green or red fluorescent ICM cells were visible in blastocysts (Supplementary Fig. S10L). Notably, examination of E13.5 embryos showed that both KLF4-rPSCs and ZFP998-rPSCs could differentiate into both somatic cells and POU5F1-expressing (GFP^+^) germ cells (Fig. 5G). The germ cell identity of GFP^+^RFP^+^ cells was verified by the expression of germ cell-specific markers (*Ddx4, Dazl*, and *Dppa3*) (Fig. 5H). We also examined the potential of ZFP998-rPSCs to maintain genomic stability. Under normal culture conditions, ZFP998-rPSCs, KLF4-rPSCs, and ESCs had comparable levels of endogenous DNA DSBs (Fig. 5I). Concordantly, they displayed similar capacities to repair DNA damage after etoposide or HU treatment (Fig. 5J and K). However, ZFP998-rPSCs, as well as KLF4-rPSCs, displayed higher rates of aneuploidy than ESCs (Supplementary Fig. S10M). This might be due to the high rate of aneuploidy in EpiSCs. Taken together, these results support that, like KLF4, ZFP998 is able to reset primed EpiSCs to naïve PSCs with high genomic stability.

### ZFP998 binds to a set of naïve and primed genes-associated enhancers and regulates their chromatin accessibility during primed-to-naïve reset

**Figure 6.**
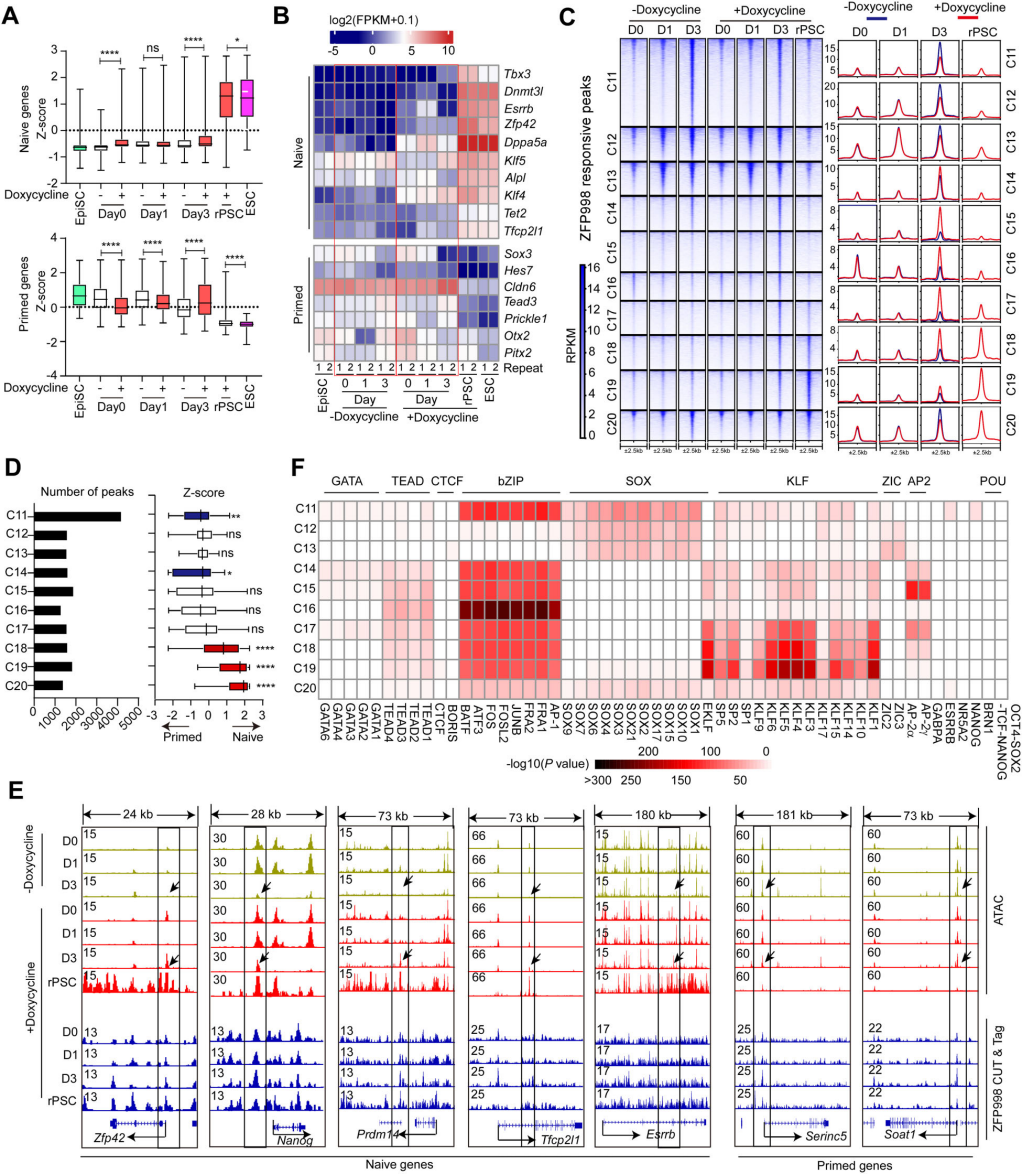
ZFP998 regulates chromatin accessibility and transcription during the primed-to-naïve pluripotency reset. (**A**) Box plots showing expression levels of defined naïve and primed gene sets at indicated time points during reprogramming, with (+doxycycline) or without (−doxycycline) ZFP998 overexpression. Naïve genes and primed genes were defined in Fig. S11B. Significance was determined using a two-sided Welch’s *t*-test. *P < 0.05, ^****^P < 0.0001; ns, not significant. (**B**) Heatmap of RNA-seq expression levels for selected naïve and primed genes during the time-course reprogramming experiment, comparing ZFP998-expressing and control conditions. Expression values are presented as log_2_ (FPKM + 0.1) to normalize variance and facilitate visualization. (**C**) Ten clusters of ATAC-seq peaks were identified that are occupied and regulated by ZFP998 (ZFP998 responsive peaks). Left panels show the heatmaps of ATAC-seq peak accessibility. Right panels show the pileups of the corresponding tag intensity. (**D**) The left panel shows the numbers of ATAC-seq peaks in each peak cluster [defined in panel (**C**)], and the right panel shows the relative expression of nearby naïve or primed genes whose TSS are located within 2500 bp of the chromatin accessibility locus. Positive *Z*-scores suggested that nearby genes were more likely to be naïve genes, whereas negative *Z*-scores indicated that nearby genes were likely to be primed genes. Significance was assessed using the Mann–Whitney U test relative to the C1 binding category. *P < 0.05, **P < 0.01, ^****^P < 0.0001; ns, not significant. (**E**) IGV tracks showing ATAC-seq signal and ZFP998 CUT&Tag occupancy at enhancer regions near key naïve (e.g. *Zfp42, Nanog, Prdm14, Tfcp2l1*, and *Esrrb*) and primed (e.g. *Serinc5, Soat1*) genes during the time course. Signal tracks are normalized as RPKM. (**F**) Motif enrichment analysis was performed for transcription factors in the ZFP998 responsive clusters of peaks [defined in (**C**)]. Color intensity reflects motif enrichment, expressed as − log_10_ (*P-*value). RNA-seq and ATAC-seq data were generated from two independent biological replicates per time point per condition.

To elucidate the molecular processes underlying ZFP998-driven reset of EpiSCs to the naïve pluripotent state, we investigated the dynamic changes of gene expression, chromatin accessibility, and ZFP998 binding on chromatin during the reset process in the presence or absence of ZFP998. Because ZFP998 drove ~10% of EpiSCs to express the GFP reporter within three days after transfer to 2i/LIF medium, we selected three time points (day 0, 1, and 3) for bulk population analysis. These time points capture the initial transcriptional events within a heterogeneous cell population, in which successfully reprogramming cells are mixed with non-responding cells. ZFP998 expression was induced by doxycycline on day 1 (Supplementary Fig. S11A), and rPSCs and ESCs were included for analyses. We first identified the gene sets that need to be activated (designated as naïve genes) or suppressed (primed genes) during cell fate conversion, by comparing transcriptomes between EpiSCs and ESCs (filtering criteria: fold change >5, FPKM > 1 in at least one cell type, *q* < 0.05) (Supplementary Fig. S11B and Supplementary Data S13). Time-course analysis of the average expression of naïve and primed genes (650 and 917 genes, respectively) revealed that ZFP998 initiated early directional shifts, upregulating naïve genes on days 0 and 3, while downregulating primed genes on days 0 and 1 (Fig. 6A). These changes, though measured in a bulk population and thus representing a diluted average signal, indicate a consistent transcriptional bias imposed by ZFP998 at the onset of reprogramming. Some critical naïve genes including *Klf4, Tbx3, Esrrb, Zfp42, Tfcp2l1, Tet2*, and *Dppa5a* (also known as *Ecat1*) were significantly up-regulated by ZFP998 (Fig. 6B). A previous study reported that intermediates during primed-to-naïve conversion acquire primitive endoderm (PrE) and trophectoderm (TE) signatures [59]. Concordantly, we found that a subset of genes upregulated by ZFP998 on day 3 (e.g. *Tgfb2, Flt1, Stxbp6, Csrp1, Fosl2, Atf3, Krt7*, and *Jun*) were known PrE/TE signature genes (Supplementary Fig. S11C).

To characterize the ZFP998-induced chromatin accessibility changes during the primed-to-naïve reset, we performed integrative analysis of ATAC-seq and ZFP998 CUT&Tag data. In total, 145 697 ATAC-seq peaks and 253 674 ZFP998 binding peaks were obtained at all time points in the presence or absence of ZFP998. A total of 62 617 overlapping ATAC-seq and ZFP998 binding peaks were identified (Supplementary Fig. S11D and Supplementary Data S14) and were classified into 20 clusters. Among them, 10 clusters of ATAC-seq peaks (C1–C10) were not influenced by ZFP998 binding (ZFP998-nonresponsive) (Supplementary Fig. S11E), whereas the remaining 10 clusters (C11-C20) showed ZFP998-dependent chromatin accessibility changes (increase or decrease, ZFP998-responsive) (Fig. 6C). Of note, the effect of ZFP998 binding on chromatin accessibility was evident only on day 3 (Fig. 6C). Intriguingly, the ZFP998-responsive peaks were predominantly localized in distal intergenic regions (Supplementary Fig. S11F), suggesting that ZFP998 might promote the primed-to-naïve transition by altering the chromatin accessibility of enhancers. Indeed, overlapping the enhancers of ESCs with ZFP998-responsive peaks revealed 1391 chromatin loci that harbor enhancers whose accessibility is regulated by ZFP998 (Supplementary Data S15).

To understand how chromatin accessibility changes influence cell state, we linked peak summits in each cluster to nearby primed or naïve genes and normalized the expression of these genes to *Z*-scores. We identified several clusters of peaks showing significant association with naïve genes (C6−C10 and C18−C20) or primed genes (C11 and C14) (Fig. 6D, Supplementary Fig. S11G, and Supplementary Data S16). Among them, naïve gene-associated peaks C18−C20 and primed gene-associated peaks C11 and C14 were ZFP998-responsive (Fig. 6D). In particular, ZFP998 increased the accessibility of peaks in C18 and C19 and suppressed the accessibility of peaks in C11 (Fig. 6C). Examples of ZFP998 binding and its regulation at enhancer peaks associated with representative naïve genes and naïve state-promoting genes (e.g. *Zfp42, Nanog, Prdm14, Esrrb, Tet2, Tfcp2l1*, and *Pou5f1*) and with primed genes (e.g. *Serinc5, Soat1, Shb*, and *Prickle1*) are shown (Fig. 6E and Supplementary Fig. S11H). Thus, ZFP998 binds to both naïve gene-associated and primed gene-associated enhancers to regulate their accessibility.

To identify other potential transcription factors involved in the primed-to-naïve resetting, we performed motif enrichment analysis on peaks from each category. Notably, motifs for the basic leucine zipper (bZIP) family and Krüppel-like factor (KLF) family transcription factors were highly enriched (Fig. 6F and Supplementary Fig. S11G), suggesting that they may collaborate with ZFP998 to regulate the resetting process.

### Coordination of naïve pluripotency and genomic stability may be conserved in human ESCs

**Figure 7.**
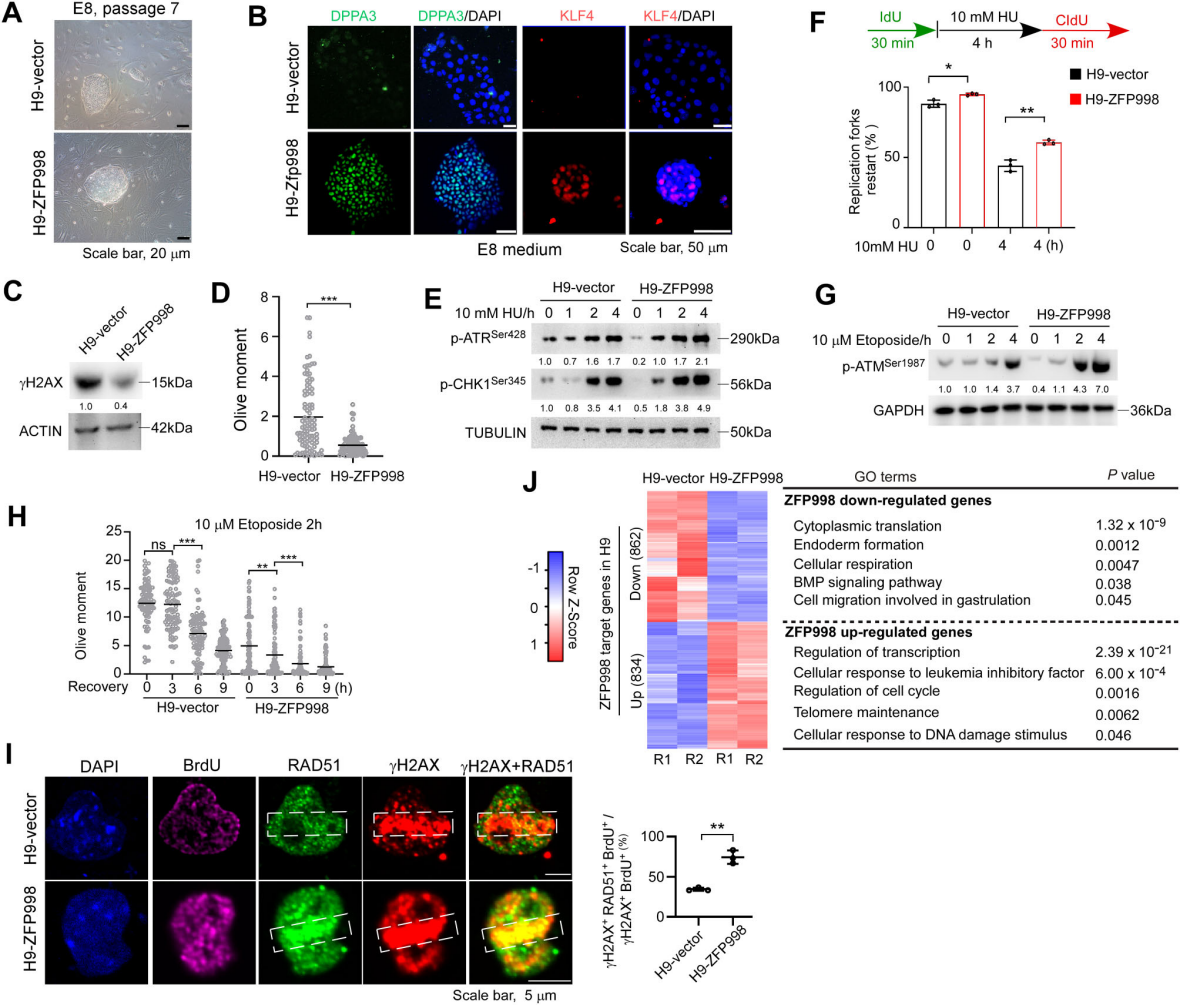
Ectopic expression of ZFP998 in H9 hESCs enhances pluripotency and genomic stability. (**A**) Representative images of H9 hESC colonies at passage 7, transduced with either a control empty vector (H9-vector) or a PiggyBac transposon vector expressing mouse ZFP998 (H9-ZFP998), and maintained in E8 medium. (**B**) Immunofluorescence staining was performed for the naïve-associated markers DPPA3 (STELLA) and KLF4 in H9-vector and H9-ZFP998 hESCs. Nuclei were counterstained with DAPI. Representative images from three independent experiments are shown. (**C**) Immunoblot analysis of γH2AX levels in H9-vector and H9-ZFP998 hESCs. ACTIN served as a loading control. (**D**) Neutral comet assay was used to quantify endogenous DNA DSBs. The tail moment was measured from at least 100 cells per group across three independent experiments. (**E**) Immunoblot analysis of the ATR-CHK1 pathway activation in H9-vector and H9-ZFP998 hESCs. Cells were treated with hydroxyurea (HU, 10 mM) for the indicated times. TUBULIN served as a loading control. (**F**) DNA fiber assay was performed to assess replication fork restart. Cells were pulse-labeled with IdU, followed by hydroxyurea (HU, 10 mM) treatment to stall replication forks. Fork restart was then monitored during a recovery period with a CldU pulse. Restart efficiency was defined as the percentage of HU-stalled forks that resumed synthesis and incorporated the second nucleotide (CldU) relative to the total number of stalled forks. Data were derived from the analysis of at least 200 fibers across three independent experiments. (**G**) Immunoblot analysis of the ATM-CHK2 pathway activation in H9-vector and H9-ZFP998 hESCs. Cells were treated with etoposide (10 µM) for the indicated times. TUBULIN served as a loading control. (**H**) DSB repair kinetics were measured by neutral comet assay. H9-vector and H9-ZFP998 hESCs were treated with etoposide (10 µM, 2 h), allowed to recover, and analyzed at the indicated times. At least 100 tails were analyzed per group. (**I**) Laser micro-irradiation assay was used to evaluate HR repair efficiency. HR repair was indicated by the co-localization of RAD51 with γH2AX in S-phase cells (BrdU^+^). H9-ZFP998 ESCs had higher HR repair efficiency than H9-vector ESCs. At least 50 cells were examined for each group. (**J**) Heatmap of RNA-seq expression for mouse ZFP998 target genes in H9-vector and H9-ZFP998 hESCs. Representative GO enrichment terms are shown for genes up- and down-regulated by ZFP998 (fold change >1.5, *P* < 0.05, *q* < 0.05). RNA-seq data are from three biological replicates. All data are presented as mean ± SEM and analyzed using two-tailed Student’s *t-*test. *P < 0.05, **P < 0.01, and ***P < 0.001. Experiments were repeated three times in independent biological replicates in (**A**-**I**).

The above studies uncovered a mechanism that coordinates naïve pluripotency and genomic stability, ensuring high differentiation potential and safety of mESCs. We wondered whether such a mechanism is conserved in humans. Zinc finger proteins evolve rapidly and no one-to-one human ortholog of mouse ZFP998 has been reported. However, if mouse ZFP998 can function in human ESCs in a similar way, this would suggest that the mechanism is conserved in humans. We thus ectopically expressed mouse ZFP998 in H9 hESCs (H9-ZFP998) (Supplementary Fig. S12A) and examined the effects. Intriguingly, compared with the control cells transfected with an empty expression vector (H9-vector), H9-ZFP998 cells cultured in the conventional E8 medium underwent morphological changes and acquired a dome-like shape 4–5 days after transfection. These dome-like hESC colonies were continuously expanded for over 20 passages (Fig. 7A). Immunofluorescence staining revealed that while DPPA3 and KLF4 were barely detected in control H9 cells, they showed enhanced immunoreactivity in H9-ZFP998 cells (Fig. 7B). This observation suggested that ectopic expression of ZFP998 induced some naïve properties in H9 hESCs. We then performed RNA-seq to comprehensively examine the gene expression changes in H9-ZFP998 cells. Expression of ZFP998 up-regulated 1342 genes (corresponding to 1358 transcripts) and down-regulated 1441 genes (corresponding to 1457 transcripts) (fold change ≥1.5, *q* < 0.05) (Supplementary Fig. S12B and Supplementary Data S17). Notably, a panel of naïve markers (e.g. *DPPA5, NR5A2, TFAP2C, UTF1, TFCP2L1, TET2*) and core DDR regulators (e.g. *ATM, ATR, RAD50, RIF1, FANCM, BRCA2*) were increased, whereas some primed pluripotency genes (e.g. *OTX2, PITX2, ZIC2, CER1, CLDN6*) were decreased (Supplementary Fig. S12C). The expression changes of several typical pluripotency and DDR genes were validated by qRT-PCR (Supplementary Fig. S12D).

The increased expression of DDR genes suggested that H9-ZFP998 cells might have an improved ability to maintain genomic stability. Indeed, H9-ZFP998 ESCs cultured in E8 medium contained fewer endogenous DNA DSBs than H9-vector cells under unperturbed conditions (Fig. 7C and D, and Supplementary Fig. S12E). In response to hydroxyurea-induced DNA replication stress, H9-ZFP998 ESCs activated ATR-CHK1 signaling more rapidly (Fig. 7E), and restarted stalled replication forks more efficiently than the H9-vector control (Fig. 7F). Similarly, upon etoposide treatment, H9-ZFP998 ESCs activated ATM signaling (Fig. 7G) and repaired DNA DSBs (Fig. 7H) more efficiently than control H9 cells. In particular, the HR-mediated repair efficiency was increased twofold in H9-ZFP998 ESCs (Fig. 7I).

We also investigated the binding sites of ZFP998 in H9 cells to understand how mouse ZFP998 regulated gene expression. Similar to the situation in mESCs, 12,455 ZFP998 peaks were detected genome-wide, and the majority (58%) were located in promoter regions in H9 cells (Supplementary Fig. S12F and Supplementary Data S18). The predicted binding motif of ZFP998 on the human genome was similar to that on the mouse genome (Supplementary Fig. S12G). Integrative analysis of RNA-seq and CUT&Tag data identified 1696 direct target genes, with ZFP998 binding at their promoters and affecting their expression (fold change ≥ 1.5) (Supplementary Data S18). Among these direct targets, 834 genes were up-regulated by ZFP998, whereas 862 genes were down-regulated (Supplementary Data S18). Consistently, the up-regulated genes were involved in processes including responses to LIF, DDR and telomere maintenance, whereas the down-regulated targets played roles in ESC differentiation (Fig. 7J and Supplementary Data S18). Representative up-regulated (e.g. *RIF1, BRCA2, RAD50, NR5A2, ATM*, and *FANCM*) (Supplementary Fig. S12H and I) and down-regulated (e.g. *ZIC2, PITX2, SOX17, EOMES*, and *OTX2*) (Supplementary Fig. S12J and K) ZFP998 target genes in H9 cells are presented.

These data indicate that ectopic expression of mouse ZFP998 in primed hESCs enhances pluripotency and genomic stability, suggesting that the coordination of pluripotency and genomic stability is conserved in human cells. Future identification of a protein with functions similar to those of mouse ZFP998 may facilitate the generation of naïve hESCs with a stable genome.

## Discussion

During *in vitro* expansion, hPSCs often acquire a wide range of genetic mutations, which may alter the cellular physiology of PSCs or their derivatives and induce oncogenic risk, thereby compromising their applications [60]. The mutation load in PSCs is tightly associated with the robustness of DDR and repair machineries. Despite enormous efforts, the expansion of human and non-human primate PSCs with full pluripotency as well as a stable genome has not been achieved. In contrast, mESCs possess both naïve pluripotency and a highly stable genome. How mESCs coordinately possess these properties remains unknown. Elucidating the underlying mechanism would facilitate the development of new strategies to generate hPSCs with full pluripotency and high genomic stability. Using mESCs as a model, we found that naïve pluripotency and high genomic stability in mESCs are coordinately regulated by a key transcription factor ZFP998. Our data support the conclusion that ZFP998 is a novel component of the naïve pluripotency regulatory circuitry. It is required not only for ESC self-renewal but also for the reprogramming from the primed state to the naïve state. In addition, it regulates the expression of a broad panel of critical DDR genes, thereby orchestrating naïve pluripotency and high genomic stability in mESCs.

Together with the known core pluripotency factors POU5F1, NANOG, SOX2, KLF4, and ESRRB, ZFP998 binds to super-enhancers in mESCs and stimulates the expression of key ESC identity genes. Depletion of ZFP998 leads to the loss of naïve pluripotency in mESCs. Moreover, POU5F1, NANOG, SOX2, KLF4, and ESRRB bind to the enhancer/promoter of *Zfp998*, and, in turn, ZFP998 occupies the enhancers/promoters of these genes and regulates their expression. ZFP998 not only maintains naïve pluripotency in mESCs but also drives the acquisition of naïve pluripotent features. Ectopic expression of ZFP998 in mouse EpiSCs reprogrammed the cells from a primed to a naïve state. ZFP998 stimulated the expression of a subset of naïve pluripotency genes, suppressed the expression of lineage commitment markers, and drove the conversion of pluripotency from primed to naïve-like state. These lines of evidence support the conclusion that ZFP998 is a novel component of the naïve pluripotency regulatory circuitry.

In addition to regulating naïve pluripotency, ZFP998 directly up-regulates the expression of many core DDR and repair genes. These DDR genes are involved in all aspects of genomic stability regulation, such as DNA replication, DNA damage signal transduction, DNA damage repair, and telomere maintenance. Thus, ZFP998 is a master regulator that ensures high genomic stability in ESCs. Unlike ZFP998, other pluripotency regulators, including POU5F1, SOX2, NANOG, and ESRRB, are unable to regulate the expression of a broad range of DDR genes [39, 56]. Although KLF4 can regulate a small number of DDR genes, its influence is far less robust compared with that of ZFP998.

The mechanistic dissection of ZFP998 function reveals a complex interplay between its distinct protein interaction modules. While the interaction with TRIM28 is canonical for KRAB domain-containing zinc finger proteins (KZFPs), our data using the ΔKRAB mutant unexpectedly showed that TRIM28 recruitment via the KRAB domain is largely dispensable for the direct transcriptional activation of core naïve pluripotency and DDR target genes measured in our rescue experiments (Fig. 4L and M). This suggests that the primary, immediate mechanism for activating these specific genes proceeds through a TRIM28-independent pathway, likely involving the DDX5/P300 axis. However, TRIM28’s essential role in reprogramming EpiSCs to the naïve state (Fig. 5B and C) indicates that this interaction is nonetheless functionally important. We propose that TRIM28 functions beyond the acute activation of individual target genes, potentially facilitating broader chromatin remodeling, maintaining epigenetic stability during the multi-step cell fate transition, or repressing alternative lineage programs that could otherwise impede reprogramming efficiency.

The interaction between ZFP998 and DDX5 is particularly intriguing. This interaction provides a direct, plausible link to explain how ZFP998 binding leads to localized histone acetylation and activation of its target genes. DDX5/DDX17 may serve as a critical adaptor or co-activator module for ZFP998, functioning to: (i) recruit the histone acetyltransferase P300 to ZFP998-bound enhancers and promoters, as supported by our interaction data; (ii) integrate transcriptional regulation with potential RNA-centric processes, as DDX5 is involved in multiple aspects of RNA metabolism and has been implicated in mediating enhancer RNA (eRNA) function or facilitating RNA polymerase II dynamics. Thus, the DDX5 interaction moves ZFP998 beyond the classical KZFP repression paradigm and positions it as a dual-function regulator capable of both TRIM28-mediated repression (potentially at a subset of loci) and DDX5/P300-mediated activation (at its characterized naïve pluripotency and DDR targets).

The zinc finger protein family evolves rapidly and represents the largest group of transcription factors in mammals [61, 62]. A clear one-to-one orthologue of mouse Zfp998 does not exist in the human genome. Nevertheless, mouse ZFP998 displayed similar functions in human cells, suggesting that the DNA motif for binding of such a transcription factor or the protein interaction network may be conserved. Based on the coupled regulatory mechanism in mESCs, in the future, it will be important to explore whether a functionally analogous factor in human cells similarly integrates the control of naïve pluripotency and genomic stability, thereby revealing conserved principles underlying cell fate regulation.

## Supplementary Material

gkag546_Supplemental_Files

## Data Availability

All data needed to evaluate the conclusions in the paper are present in the paper and/or the Supplementary Materials. The raw data for mouse RNA-seq, CUT&tag, and ATAC-seq have been deposited in the National Genomics Data Center database (https://ngdc.cncb.ac.cn/gsa/) with the following accession numbers: CRA017458. Human ESCs RNA-seq and CUT&tag data have been deposited in the National Genomics Data Center database (https://ngdc.cncb.ac.cn/gsa-human/) with the following accession numbers: HRA007811. The raw data of MS in Fig. 4A were deposited to the ProteomeXchange Consortium (http://proteomecentral.proteomexchange.org) via the iProX partner repository with the dataset identifier PXD071448.
