# Supplementary material for: Naïve pluripotency and genomic stability are coordinated in embryonic stem cells by a novel pluripotency regulator ZFP998
Source: Nucleic Acids Res. 2026 May 27;54(10):gkag546. doi: 10.1093/nar/gkag546 (PMC13213254; doi:10.1093/nar/gkag546)
Supplement: gkag546_Supplemental_Files [file gkag546_supplemental_files.zip › 220526064504_Supplementary_Materials_.pdf]

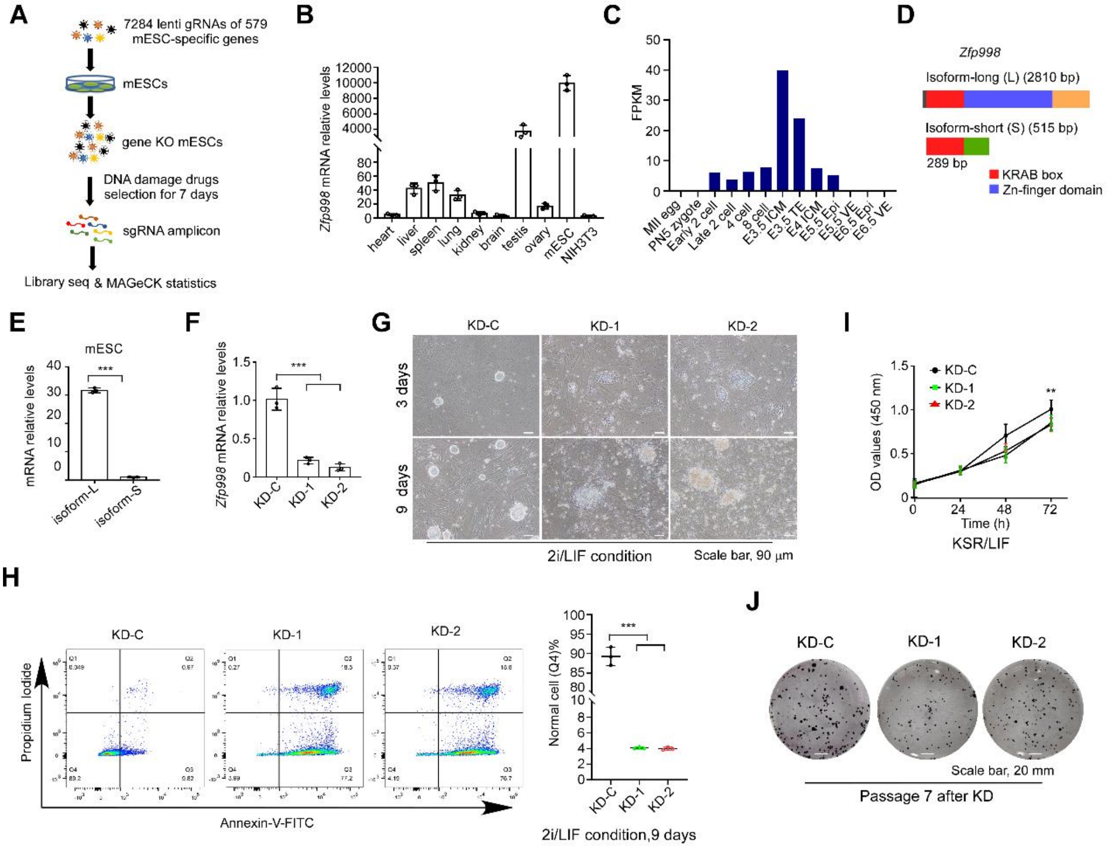

Supplemental Figure 1: Identification and characterization of *Zfp998* knockdown in mESCs.

(**A**) Schematic of the pooled CRISPR/Cas9 screening workflow to identify regulators of genomic stability. A custom sgRNA library targeting 579 mESC-specific genes was cloned into a lentiviral vector. The library was transduced at a low multiplicity of infection (MOI = 0.3) into a Cas9-expressing mESC line to ensure single integrations per cell. After puromycin selection, cells were cultured for 7 days in the presence of one of seven DNA-damaging agents: etoposide (20 nM, topoisomerase II inhibitor), hydroxyurea (200 nM, DNA synthesis inhibitor), melphalan (200 nM, DNA crosslinker), olaparib (25 nM, PARP inhibitor), 6-mercaptopurine (200 nM, purine analogue), topotecan (50 nM, topoisomerase I inhibitor), or zeocin (25 nM, DNA-cleaving agent). Genomic DNA was harvested from the initial transduced pool (T0) and the final population (T7). sgRNAs were amplified and subjected to next-generation sequencing. sgRNA abundance was quantified, and gene-level enrichment or depletion scores were calculated using the MAGeCK algorithm (v0.5.9.2) with default parameters.

(**B**) Tissue distribution of *Zfp998* expression. *Zfp998* mRNA levels were quantified by qRT-PCR using isoform-long-specific primers. Expression was normalized to *Actb*. The heart sample was set to 1. Data points represent mean ± SEM from three independent RNA preparations.

(**C**) Developmental expression profile of *Zfp998* during early embryogenesis. MII oocyte, PN5 zygote, 2-cell early, 2-cell late, 4-cell, and 8-cell stage data were obtained from GSE71434 (PMID: 27626382). Data for E3.5 ICM, E4, E5.5, and E6.5 stages were from GSE76505 (PMID: 29203909).

(**D**) Coding sequence organization of the two transcript isoforms of *Zfp998*. Schematic of the mouse *Zfp998* gene locus illustrating the two major transcript isoforms (2,810 bp and 515 bp). Exons are represented by colored boxes, with identical colors indicating identical nucleotide sequences. The diagram is based on the RefSeq annotation (mm10).

(**E**) Isoform-specific expression analysis in mESCs. Isoform-specific qRT-PCR was performed using primers that uniquely amplify either the long (2,810 bp) or short (515 bp) isoform, with normalization to *Actb*. The short isoform was set to 1. Data are presented as mean ± SEM from three independent experiments.

(**F**) Validation of *Zfp998* knockdown (KD) efficiency. mESCs were transduced with lentiviral vectors expressing one of two independent shRNAs targeting *Zfp998* (KD-1, KD-2) or a non-targeting control shRNA (KD-C). *Zfp998* mRNA levels were quantified by qRT-PCR (primers as in B). Expression was normalized to *Actb*. KD-C was set to 1. Data are presented as mean ± SEM from three independent transductions.

(**G**) Cell morphology of KD-C and *Zfp998* KD mESCs cultured under 2i/LIF condition for 3 days or 9 days. KD-C and *Zfp998* KD mESCs were seeded at equal density (200 cells/cm^2) in 2i/LIF medium. Representative images were acquired after 3 and 9 days of culture using a 10× objective. KD-C cells maintained compact, dome-shaped colonies, whereas *Zfp998* KD colonies progressively flattened and dispersed.

(**H**) Flow cytometry analysis of apoptosis and necrosis using Annexin V-FITC/PI staining in third-passage (9 days) KD-C and *Zfp998* KD ESCs cultured in 2i/LIF. Left panel, representative dot plots showing Annexin V-FITC (x-axis) and PI (y-axis) staining in KD-C and *Zfp998* KD ESCs, with quadrants indicating normal (Q4: Annexin V^-/PI^-), early apoptotic (Q3: Annexin V^+/PI^-), late apoptotic/necrotic (Q2: Annexin V^+/PI^+), and necrotic (Q1: Annexin V^-/PI^+) populations. Right panel, quantification of normal cells (Q4) as a percentage of total cells. *Zfp998* KD led to a significant reduction in the normal cell population compared with KD-C. Data are presented as mean ± SEM from three independent experiments.

(**I**) KD-C and *Zfp998* KD mESCs were seeded in triplicate 96-well plates (2,000 cells/well). At 24, 48, and 72 h, 10 µL of CCK-8 reagent was added per well. After 2-h incubation at 37°C, absorbance was read at 450 nm. Data are presented as mean ± SEM from three independent experiments, with six replicate wells per condition in each experiment.

(**J**) AP staining of KD-C and *Zfp998* KD mESCs at passage 7. Colonies retaining alkaline phosphatase activity stain red/purple (AP^+). Representative images from three independent experiments are shown.

All experiments in B and E-J were performed in three independent biological replicates. Data are presented as mean ± SEM and analyzed using two-tailed Student’s *t*-test. $^{**}P < 0.01$, $^{***}P < 0.001$.

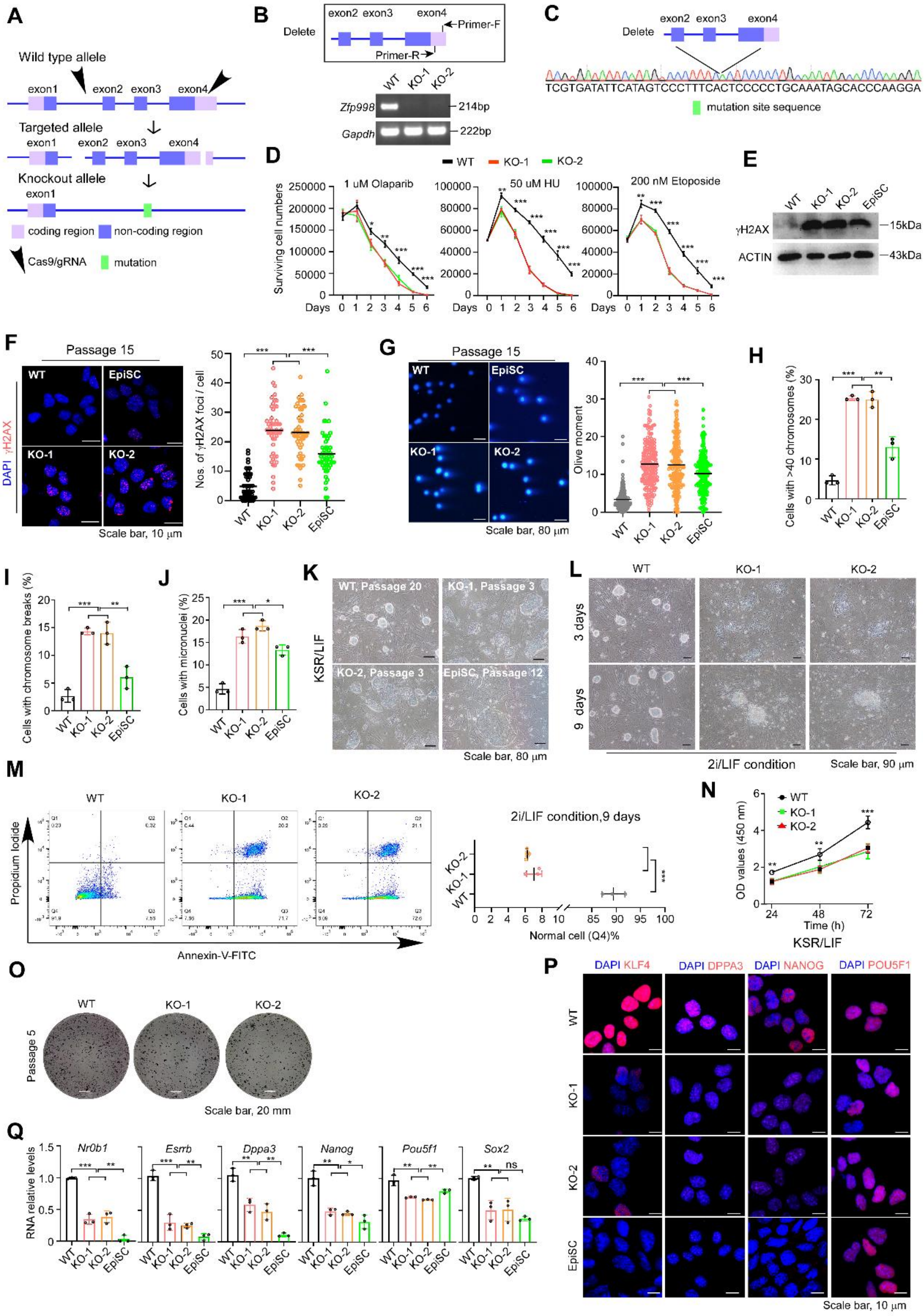

Supplemental Figure 2: Genomic instability and pluripotency defects in *Zfp998* knockout mESCs.

(**A**) Strategy for generating *Zfp998* knockout (KO) mESCs using CRISPR-Cas9.

(**B** and **C**) Genotypic validation of *Zfp998* knockout (KO) mESCs. **(B)** RT-PCR analysis of genomic DNA from wild-type (WT) and KO mESCs using primers flanking the CRISPR-targeted region. The primer pair yields an amplicon in WT cells but no product in the KO clone, confirming deletion of the targeted genomic segment.
(C) Sanger sequencing chromatograms of genomic PCR products amplified with primers spanning the targeted region, confirming the loss of the corresponding DNA sequence in the KO clone.

(**D**) Drug sensitivity assay. WT and *Zfp998* KO mESCs were treated for 72 hours with Olaparib (1 μM, left panel), hydroxyurea (HU, 50 μM, middle panel), or etoposide (200 nM, right panel). Viability was assessed by direct cell counting.

(**E** and **F**) The γH2AX levels was detected by Western blot (**E**) and immunofluorescent staining (**F**) in WT mESCs, *Zfp998* KO mESCs and EpiSCs. In F, representative images are shown in the left panel. Quantification of the mean number of γH2AX foci per nucleus is shown in the right panel. At least 30 fields of view containing 200 cells per condition were analyzed.

(**G**) Neutral comet assay showing endogenous DSB levels in WT mESCs, *Zfp998* KO mESCs, and EpiSCs. Representative images are shown in the left panel. Quantification of the tail moment is shown in the right panel. At least 200 cells were analyzed per group.

(**H-J**) Frequency of aneuploidy (**H**), chromosome breakage (**I**), and micronuclei (**J**) in WT mESCs, *Zfp998* KO mESCs, and EpiSCs. For (H) and (I), at least 50 metaphase spreads in three independent replicates were analyzed. For (J), at least 50 visual fields per group, containing 1,000 cells, were analyzed in three replicates.

(**K**) Cell colony morphology of WT mESCs, *Zfp998* KO mESCs, and EpiSCs. ESCs were cultured in KSR/LIF medium. Representative images are shown.

(**L**) Cell colony morphology of WT and *Zfp998* KO mESCs cultured in 2i/LIF medium for 3 days or 9 days. Representative images are shown.

(**M**) Apoptosis assay for *Zfp998* KO cells. Annexin V-FITC/PI staining was performed in third-passage WT and *Zfp998* KO ESCs cultured in 2i/LIF for 9 days. Representative FACS results are shown in the left panel. Quantification of live cells is shown in the right panel. Data are presented as mean ± SEM from three independent experiments.

(**N**) Cell proliferation assay. Viability was measured at 24, 48, and 72 hours using the CCK-8 assay (absorbance at 450 nm).

(**O**) *Zfp998* KO mESCs were positive for alkaline phosphatase staining (AP^+). Representative images from three independent experiments are shown.

(**P**) Representative images of naïve (KLF4 and DPPA3) and core (NANOG and POU5F1) pluripotency marker staining in WT mESCs, *Zfp998* KO mESCs, and EpiSCs. Nuclei were counterstained with DAPI.

(**Q**) Quantitative RT-PCR analysis of naïve (e.g., *Nr0b1*, *Esrrb*, *Dppa3*) and core (e.g., *Pou5f1*, *Sox2*, *Nanog*) pluripotency marker gene expression in WT mESCs, *Zfp998* KO mESCs, and EpiSCs. Expression levels were normalized to *Actb*. WT was set as 1.

All data are presented as mean ± SEM and analyzed using two-tailed Student's *t*-test. *$P < 0.05$, **$P < 0.01$, ***$P < 0.001$. All experiments were repeated at least three times using independent biological samples.

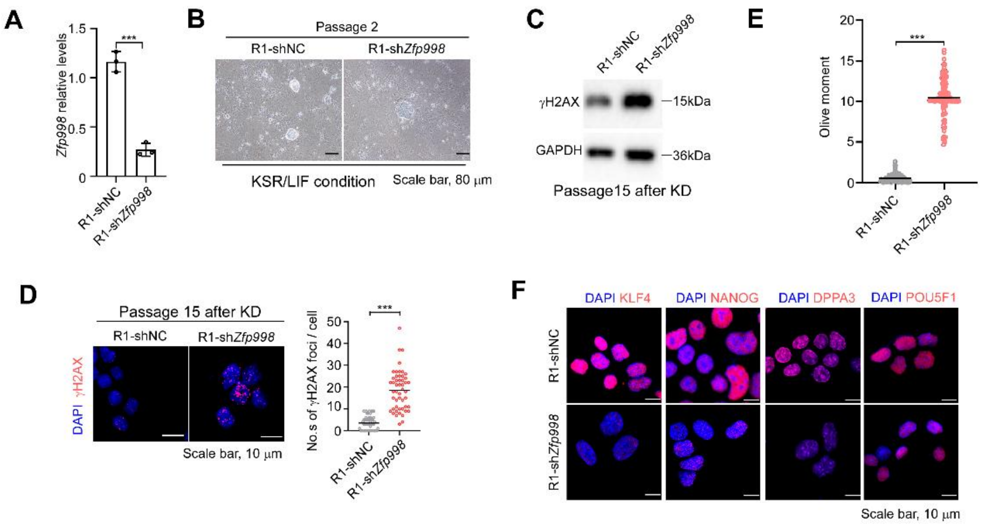

Supplemental Figure 3: ZFP998 regulates the expression of pluripotency and DDR genes in the R1 mESC line.

(A) Validation of *Zfp998* knockdown efficiency. R1 mESCs were transduced with lentivirus expressing a short hairpin RNA targeting *Zfp998* (R1-sh*Zfp998*) or a non-targeting control shRNA (R1-shNC, negative control). *Zfp998* mRNA levels were quantified by quantitative RT-PCR using *Zfp998*-specific primers and normalized to *Actb*. R1-shNC was set to 1.

(B) Representative images of R1-shNC and R1-sh*Zfp998* mESCs were captured after 5 days of culture using a 10 × objective lens.

(C) Immunoblot analysis of γH2AX protein levels in R1-shNC and R1-sh*Zfp998* mESCs. GAPDH served as a loading control.

(D) Immunostaining of γH2AX was peformed in *Zfp998* KD R1 cells. Representative images are shown in the left panel. Quantification of the mean number of γH2AX foci per nucleus is shown in the right panel. At least 30 fields of view containing 200 cells were examined for R1-shNC and R1-sh*Zfp998* mESCs.

(E) Neutral comet assay was used to quantify endogenous DNA double-strand breaks (DSBs) in R1-shNC and R1-sh*Zfp998* mESCs. At least 200 cells were analyzed per group.

(F) R1-shNC and R1-sh*Zfp998* mESCs were fixed and stained with antibodies against the naïve pluripotency markers KLF4 and DPPA3, and the core pluripotency markers NANOG and POU5F1. Nuclei were counterstained with DAPI. Representative images are shown.

All data are presented as mean ± SEM and analyzed using two-tailed Student's *t*-test, $^{**}P < 0.001$. All experiments were repeated at least three times using independent biological samples.

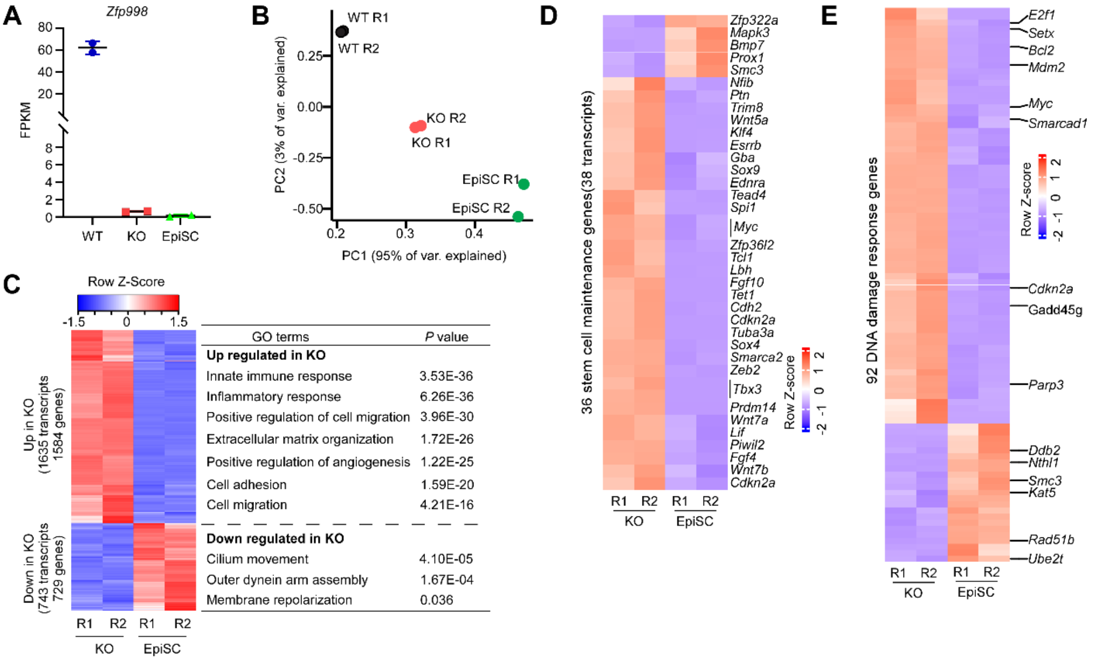

Supplemental Figure 4: *Zfp998* KO downregulates the expression of pluripotency and DDR genes.

(A) Transcript abundance of *Zfp998* was determined using RNA-seq data from wild-type (WT) mESCs, *Zfp998* knockout (KO) mESCs, and EpiSCs. Two independent biological replicates were analyzed per group. Expression levels are presented as fragments per kilobase of transcript per million mapped reads (FPKM).

(B) Principal component analysis (PCA) of WT, *Zfp998* KO mESCs, and EpiSCs. Percentages indicate the proportion of variance explained by each principal component (PC1 and PC2).

(C) Differentially expressed genes (DEGs) were identified from comparison between *Zfp998* KO and EpiSCs. Heatmaps of DEGs are shown in the left panel, and corresponding Gene Ontology (GO) enrichment analysis of DEGs is shown in the right panel.

(D and E) Heatmaps display expression patterns of representative pluripotency **(D)** and DDR **(E)** genes in *Zfp998* KO mESCs and EpiSCs.

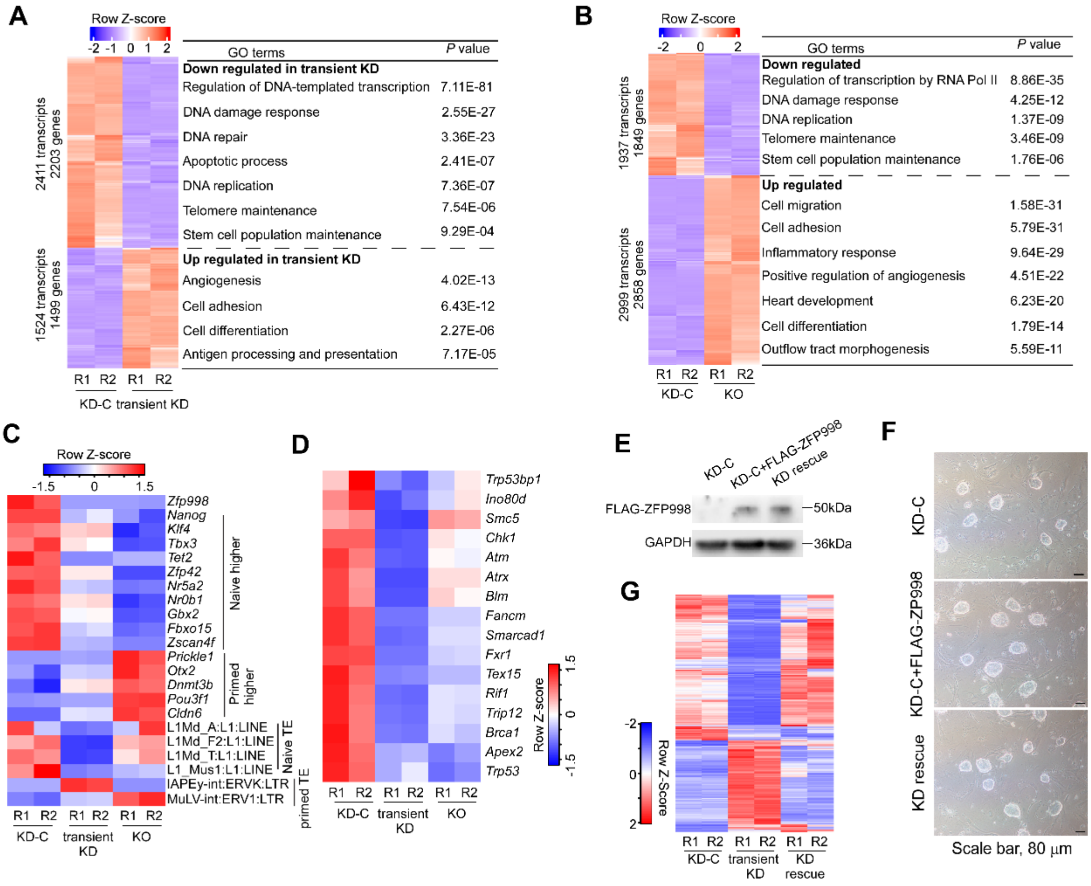

Supplemental Figure 5: ZFP998 regulates the expression of pluripotency and DDR genes.

(A) Heatmap of differentially expressed genes (DEGs) between knockdown control (KD-C) and transient *Zfp998* KD mESCs is shown in the left panel, with corresponding Gene Ontology (GO) enrichment terms shown in the right panel.

(B) Heatmap of DEGs between KD-C and *Zfp998* KO mESCs is shown in the left panel, with corresponding GO enrichment terms shown in the right panel.

(C and D) Heatmaps of representative pluripotency **(C)** and DDR **(D)** gene expression profiles among KD-C, transient *Zfp998* KD, and *Zfp998* KO mESCs.

(E) Immunoblot validation of FLAG-ZFP998 re-expression in rescue experiments. GAPDH served as a loading control. Three independent experiments were performed.

(F) Re-expression of FLAG-ZFP998 in the KD background (KD rescue) restored the compact, dome-shaped colony morphology, indistinguishable from that of KD-C and KD-C expressing FLAG-ZFP998. Three independent experiments were performed.

(G) Heatmap of DEGs shows their expression patterns under KD-C, transient KD, and KD rescue conditions.

All analyses were performed using two biological replicates in A-D and G.

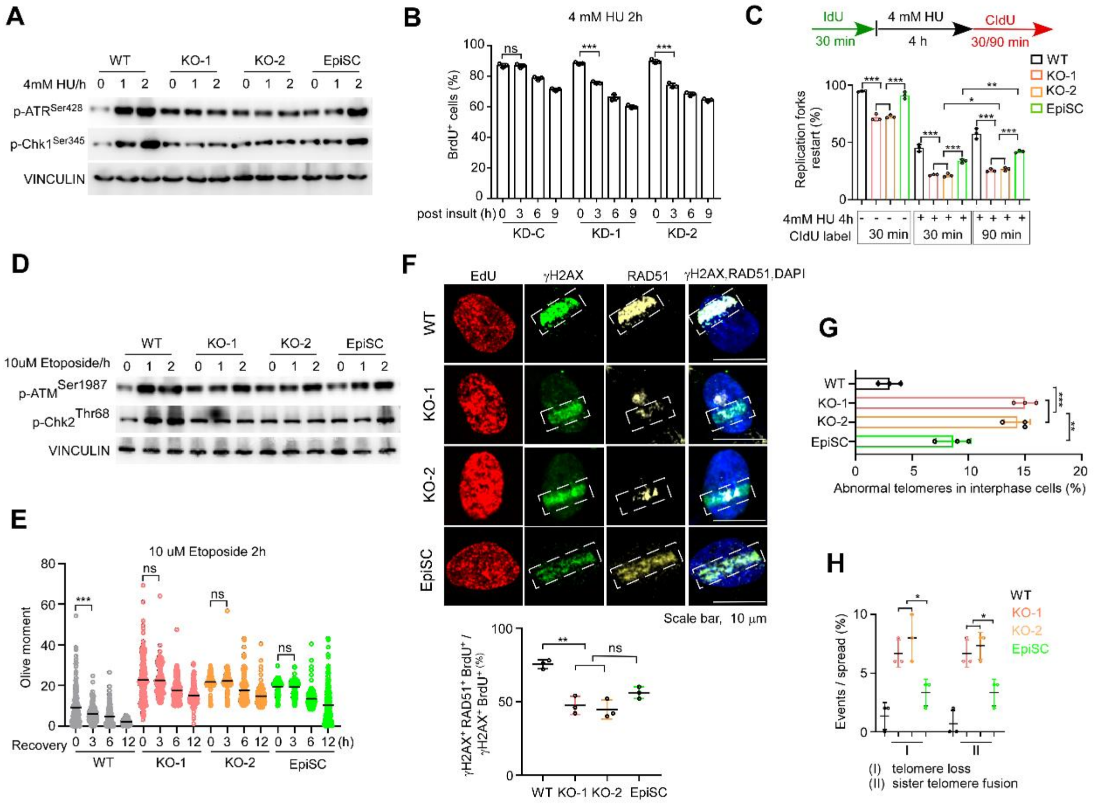

Supplemental Figure 6: *Zfp998* KO mESCs exhibit impaired DNA damage response and repair.

(A) Immunoblot analysis of the ATR-CHK1 signaling pathway. WT mESCs, *Zfp998* KO mESCs, and EpiSCs were treated with hydroxyurea (HU, 4 mM) for the indicated times. VINCULIN served as a loading control.

(B) Cell cycle analysis by BrdU incorporation. The intra-S-phase checkpoint was assessed by monitoring S-phase progression after replication stress. Cells were treated with 4 mM hydroxyurea (HU) for 2 hours, allowed to recover for the indicated times, and then pulse-labeled with BrdU (10 μM) for 10 minutes to mark cells in active DNA synthesis. The percentage of BrdU-positive (S-phase) cells was quantified by flow cytometry. Compared with KD-C, *Zfp998* KD mESCs exhibited a persistently lower proportion of S-phase cells during recovery, indicating a potential deficiency in the activation or recovery from the intra-S-phase checkpoint.

(C) Replication fork restart abilities in WT mESCs, *Zfp998* KO mESCs, and EpiSCs following HU treatment, assessed by DNA fiber assay. At least 200 fibers from three independent experiments were analyzed.

(D) Immunoblot analysis of the ATM-CHK2 signaling pathway. WT, *Zfp998* KO mESCs, and EpiSCs were treated with etoposide (10 μM) for the indicated times. VINCULIN served as a loading control.

(E) DNA DSB repair abilities in WT mESCs, *Zfp998* KO mESCs, and EpiSCs, as detected by neutral comet assay. At least 200 cells were analyzed per group.

(F) HR-mediated DNA DSB repair efficiency in WT mESCs, *Zfp998* KO mESCs, and EpiSCs measured by laser micro-irradiation assay. Representative images are shown in the top panel. Quantification of DSB repair efficiency is shown in the lower panel. At least 50 cells were examined per group.

(G and H) Rates of abnormal telomeres in WT mESCs, *Zfp998* KO mESCs, and EpiSCs at interphase **(G)** and metaphase **(H)**, as revealed by telomere FISH. At least 50 cells or 50 metaphase spreads were examined per group.

All data are presented as mean ± SEM and analyzed using two-tailed Student's *t*-test. $^{**}P < 0.01$, and $^{***}P < 0.001$. Experiments were repeated at least three times using independent biological samples.

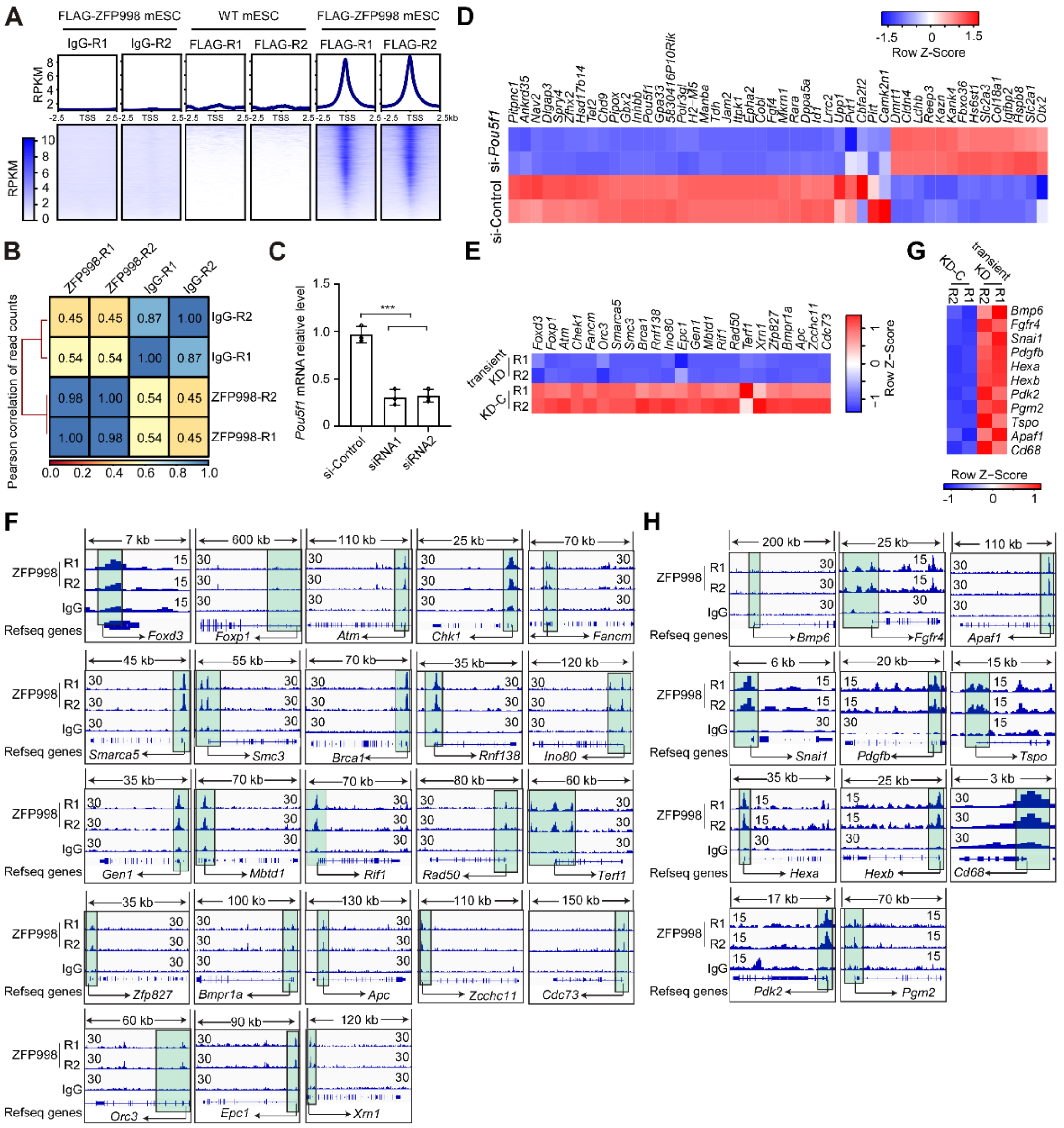

Supplemental Figure 7: ZFP998 binds to enhancers and promoters of DDR and stem cell maintenance genes.

(A) Heatmaps showing CUT&Tag signals at promoters (±2.5 kb from TSS) in mESCs expressing FLAG-ZFP998 (IgG control, left panel; FLAG, right panel) and in wild-type (WT) mESCs (FLAG, middle panel).

(B) Reproducibility of ZFP998 CUT&Tag replicates. Pearson correlation coefficients were calculated based on genome-wide read counts in 10-kb bins for two independent biological replicates (R1 and R2).

(C) Validation of *Pou5f1* knockdown efficiency. mESCs were transfected with two independent siRNAs targeting *Pou5f1* (siRNA1 and siRNA2) or a non-targeting control siRNA (si-Control). *Pou5f1* mRNA levels were quantified by quantitative RT-PCR at 72 hours post-transfection. Expression was normalized to *Actb*. si-Control was set to 1. Data are presented as mean ± SEM from three independent

transfections, each assayed in technical triplicate. Statistical significance was determined by a two-tailed Student's *t*-test (***$P < 0.001$).

(D) Heatmap of mESC identity gene expression upon *Pou5f1* knockdown. Data are derived from two independent biological replicates per condition (si-Control and si-*Pou5f1* pools).

(E) Heatmap of selected ZFP998-directly-activated target genes, with data derived from two biological replicates per condition.

(F) Integrative Genomics Viewer (IGV) browser tracks showing ZFP998 binding at the promoter regions of the activated target genes listed in **(E)**.

(G) Heatmap of selected ZFP998-directly-suppressed target genes. Genes whose promoters are bound by ZFP998 and whose expression is downregulated upon transient *Zfp998* knockdown are defined as ZFP998-directly-suppressed genes. Data are from two biological replicates per condition.

(H) Integrative Genomics Viewer (IGV) browser tracks showing ZFP998 binding at the promoter regions of the suppressed target genes listed in **(G)**.

All experiments were performed with at least two independent biological replicates, with the exception of the experiment in C, which was repeated three times.

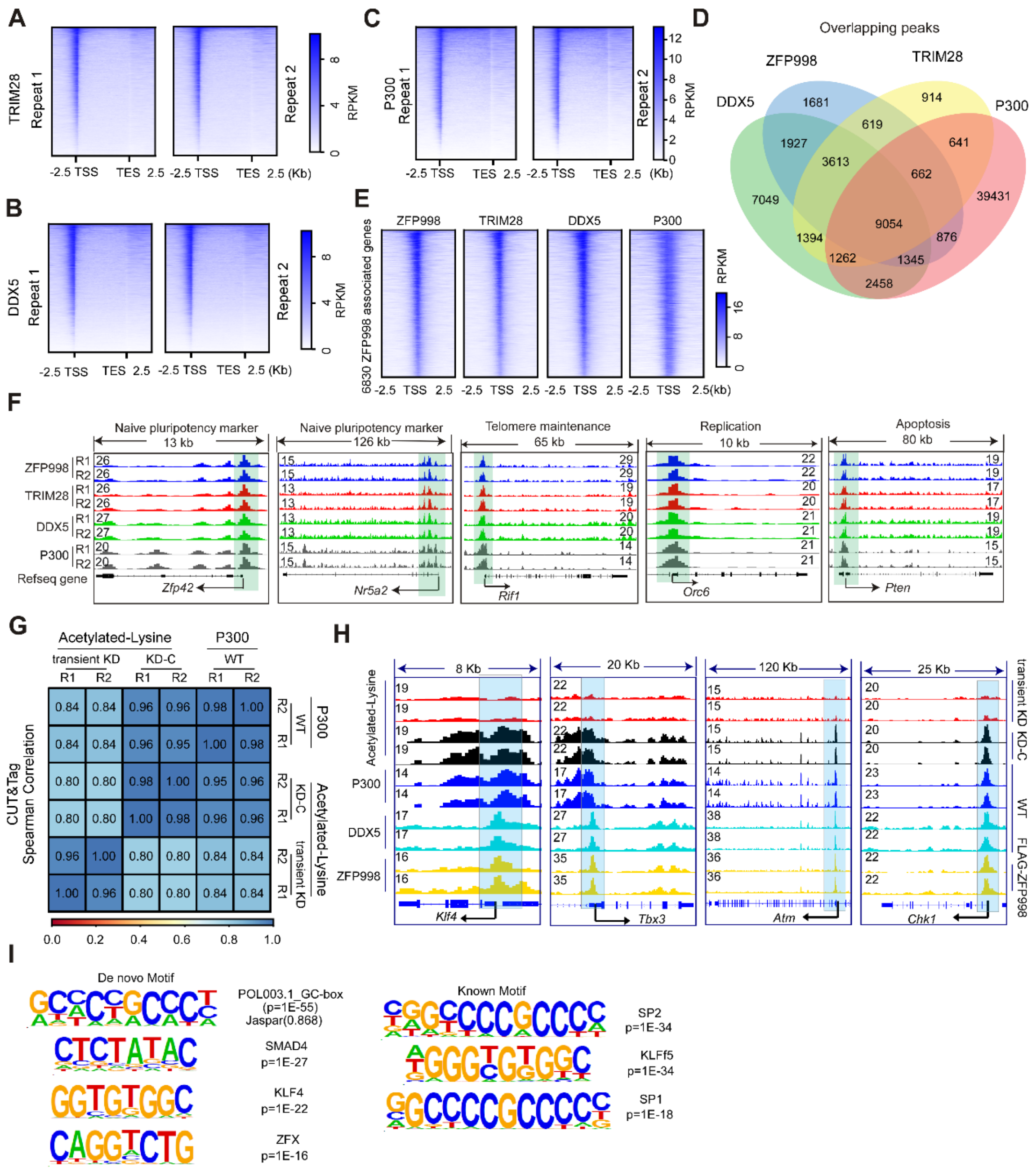

Supplemental Figure 8: ZFP998 interacts with DDX5, TRIM28, and P300.

(A–C) Genome-wide binding profiles of **(A)** TRIM28, **(B)** DDX5, and **(C)** P300 in mESCs. Heatmaps display tag intensity of TRIM28, DDX5, and P300 around gene bodies for two independent biological replicates. TSS, transcription start site; TES, transcription end site.

(D) Venn diagrams illustrate the overlap between ZFP998 (blue), DDX5 (green), TRIM28 (yellow), and P300 (red) peaks, called using MACS2 ($q < 0.05$).

(E) Heatmaps show CUT&Tag signals for TRIM28, DDX5, and P300 at promoters (±2.5 kb from TSS) of ZFP998-associated genes (n = 9,154).

(F) Integrative Genomics Viewer (IGV) browser tracks show co-occupancy of ZFP998, TRIM28, DDX5, and P300 at promoters of representative naïve pluripotency (*Zfp42*, *Nr5a2*) and DDR (*Rif1*, *Orc6*, and *Pten*) genes. Signals are normalized as RPKM.

(G) Correlation analysis of Acetylated-Lysine and P300 CUT&Tag replicates. Spearman correlation coefficients were calculated based on genome wide read counts in 10 kb bins for two independent biological replicates (R1 and R2).

(H) IGV browser tracks show the co-localization of ZFP998, TRIM28, DDX5, and P300 at promoters of key naïve pluripotency genes (*Tbx3* and *Klf4*) and DDR genes (*Chk1* and *Atm*), where acetylation signal intensity is decreased following transient *Zfp998* KD.

(I) Motif enrichment analysis of ZFP998 binding sites was performed. *De novo* motif enrichment analysis is shown in the top panel, and known motif enrichment analysis in the bottom panel.

All experiments were performed with two independent biological replicates.

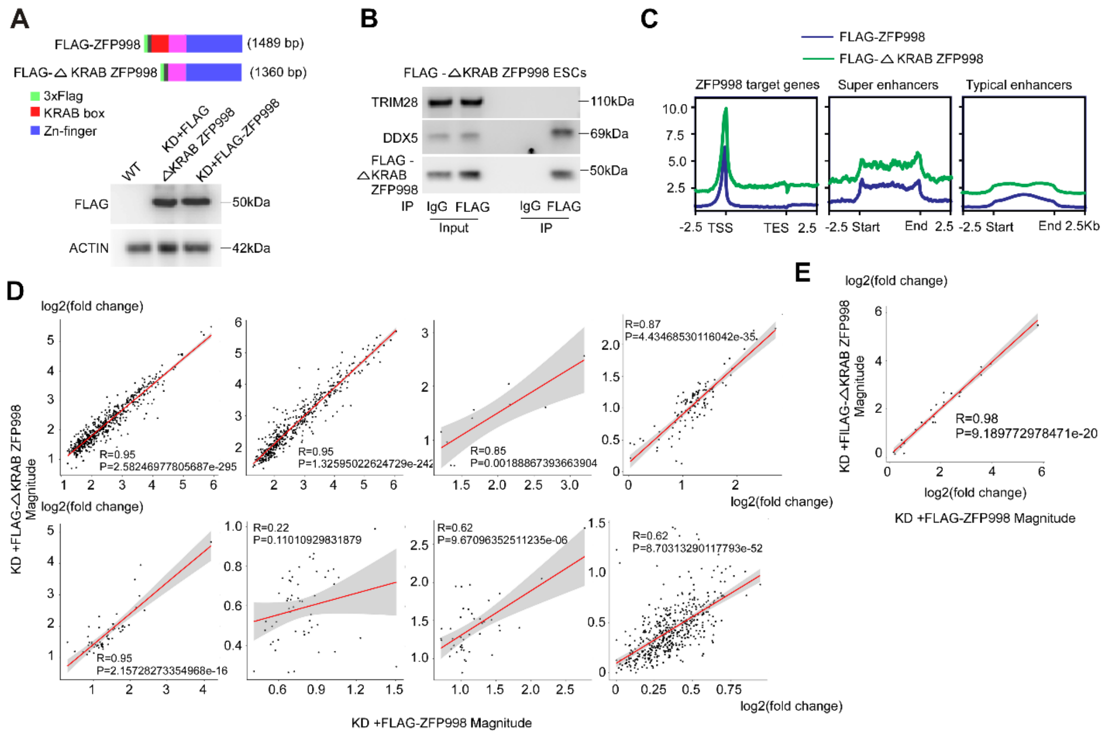

Supplemental Figure 9: ZFP998 regulates gene expression independently of its KRAB domain.

(A) Generation of an mESC line expressing FLAG-tagged ΔKRAB ZFP998 protein. *Zfp998* KD mESCs were transduced with a PiggyBac transposon vector carrying a ΔKRAB *Zfp998*-FLAG expression cassette. Stable integrants were selected, and expression was confirmed by immunoblotting with an anti-FLAG antibody. Three independent experiments were performed.

(B) Validation of the interaction between mutant ZFP998 and TRIM28 or DDX5. Co-immunoprecipitation (co-IP) was performed using an anti-FLAG antibody, with normal IgG immunoprecipitation serving as the negative control. Immunoblotting confirmed that ΔKRAB ZFP998 failed to co-precipitate TRIM28 but retained interaction with DDX5. Input (5%) and IP fractions from both the anti-FLAG and control IgG pull-downs are shown. Three independent experiments were performed.

(C) Profile plots showing CUT&Tag signals for wild-type (blue) and ΔKRAB ZFP998 (green) at typical enhancers, super-enhancers, and gene bodies of ZFP998 target genes.

(D) Correlation analysis of gene expression rescue between wild-type ZFP998 (KD + FLAG-ZFP998) and the ΔKRAB mutant (KD + FLAG-ΔKRAB ZFP998) (related to Fig. 4J). X-axis: $\log_2$ ((KD + FLAG-ZFP998 + 0.1) / (transient KD + 0.1)), representing expression changes upon wild-type ZFP998 rescue. Y-axis: $\log_2$ ((KD + FLAG-ΔKRAB ZFP998 + 0.1) / (transient KD + 0.1)), representing expression changes upon

ΔKRAB rescue. A constant of 0.1 was added to all values to avoid division by zero and to stabilize the calculation.

(E) Correlation analysis of gene expression rescue magnitude between wild-type ZFP998 and the ΔKRAB mutant (related to Fig. 4K). Axes are defined as in (D).

Experiments in C-E were performed with at least two independent biological replicates.

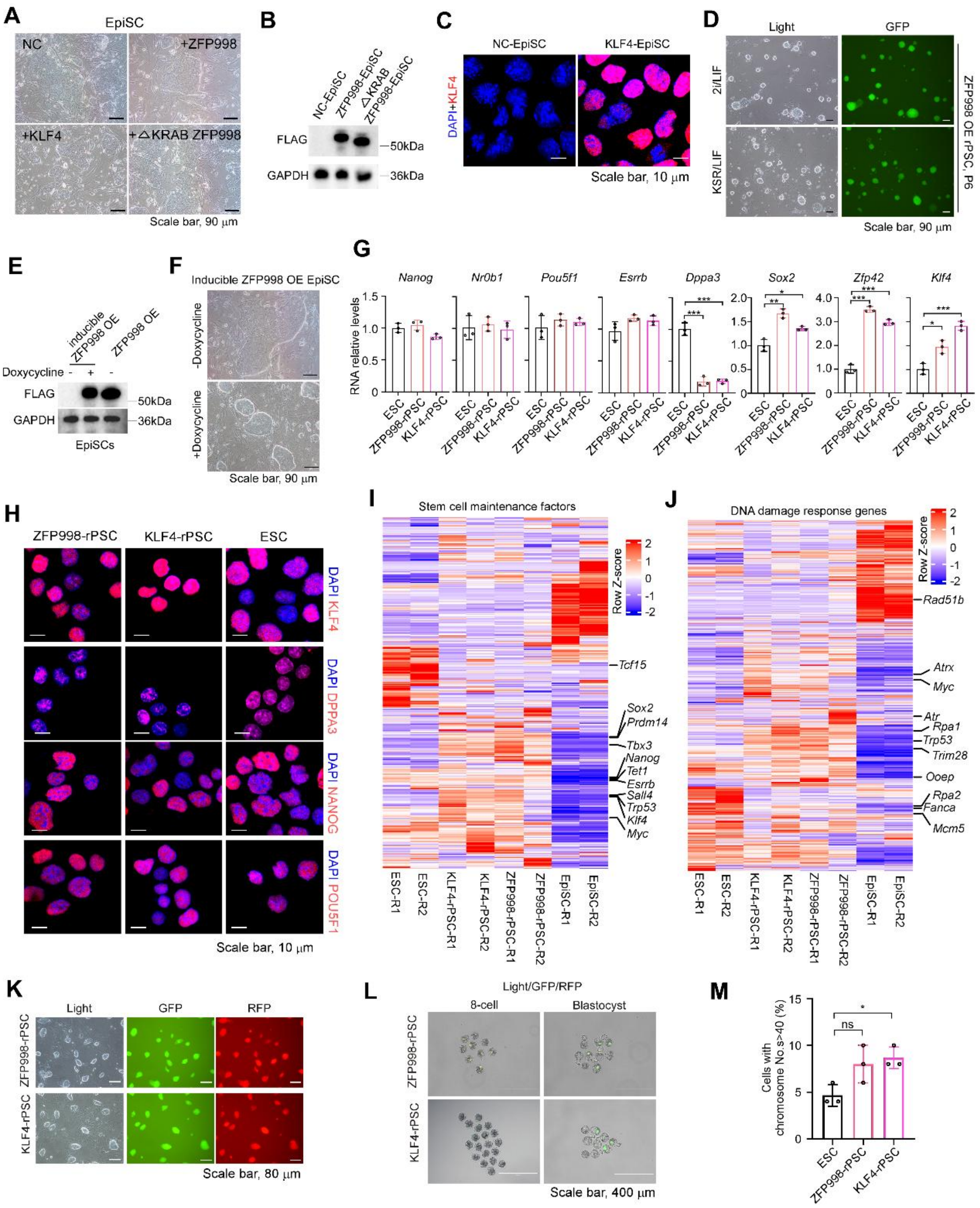

Supplemental Figure 10: Reprogramming of primed epiblast stem cells (EpiSCs) to naïve pluripotency by ZFP998.

(A) Morphology of EpiSC colonies expressing control vector (NC), ZFP998, KLF4, or ΔKRAB ZFP998. EpiSCs transduced with vectors expressing control (NC), wild-type ZFP998, KLF4, or ΔKRAB ZFP998 were cultured in FA medium. Representative images are shown.

(B) Immunoblot analysis using an anti-FLAG antibody confirms the expression of FLAG-tagged ZFP998 and ΔKRAB ZFP998 in transduced EpiSCs. GAPDH served as a loading control. Three independent experiments were performed.

(C) Immunofluorescence staining for KLF4 confirms its expression in KLF4-transduced EpiSCs (KLF4-EpiSC). Nuclei were counterstained with DAPI.

(D) Stable ZFP998-induced rPSCs were cultured in either 2i/LIF or KSR/LIF medium at passage 6. Phase-contrast images show naïve colony morphology, and fluorescence images show sustained *Pou5f1*-GFP reporter expression.

(E) Generation of a stable EpiSC line with doxycycline-inducible ZFP998 expression. EpiSCs were transduced with PiggyBac transposon vectors carrying a doxycycline-inducible cassette for wild-type ZFP998 expression. Stable integrants were selected with puromycin, and single-cell clone lines were established. Inducible expression was validated by immunoblotting after doxycycline treatment. Three independent experiments were performed.

(F) Representative images of EpiSCs with or without doxycycline-induced ZFP998 expression.

(G) Expression of naïve (*Nr0b1*, *Esrrb*, *Dppa3*, *Zfp42*, *Klf4*) and core pluripotency (*Nanog*, *Pou5f1*, *Sox2*) genes in wild-type mESCs, ZFP998-rPSCs, and KLF4-rPSCs, detected by quantitative RT-PCR. RNA expression was normalized to *Actb*. mESCs were set to 1. Data are presented as mean ± SEM from three independent experiments and analyzed using two-tailed Student's t-test. *$P < 0.05$, **$P < 0.01$, ***$P < 0.001$.

(H) Immunofluorescence analysis of pluripotency markers. Cells from **(G)** were fixed and stained for KLF4, DPPA3, POU5F1, and NANOG. Nuclei were counterstained with DAPI. Representative images are shown. Three independent experiments were performed.

(I and J) **(I)** Heatmap of stem cell maintenance genes among ESCs, KLF4-rPSCs, ZFP998-rPSCs, and EpiSCs. **(J)** Heatmap of DNA damage response genes among ESCs, KLF4-rPSCs, ZFP998-rPSCs, and EpiSCs. Data are from two biological replicates per cell type. Stem cell maintenance genes and DNA damage response genes were obtained from AmiGO.

(K) ZFP998-rPSCs and KLF4-rPSCs were transduced with an RFP-expressing lentivirus. Representative bright-field (Light) and fluorescent (GFP and RFP) images of ZFP998-rPSCs and KLF4-rPSCs are shown.

(L) KLF4-rPSCs and ZFP998-rPSCs were injected into 8-cell mouse embryos (4–6 cells/embryo). Inner cell mass cells of blastocysts co-expressed RFP and GFP.

(M) Aneuploidy rates in ESCs, KLF4-rPSCs, and ZFP998-rPSCs. Metaphase spreads were prepared from mESCs, KLF4-rPSCs, and ZFP998-rPSCs. The frequency of aneuploid metaphases (chromosome number ≠ 40) was determined by

scoring at least 50 spreads per cell line across three independent experiments. Data are presented as mean ± SEM from three independent biological replicates and analyzed using two-tailed Student's *t*-test. *$P < 0.05$; ns, not significant.

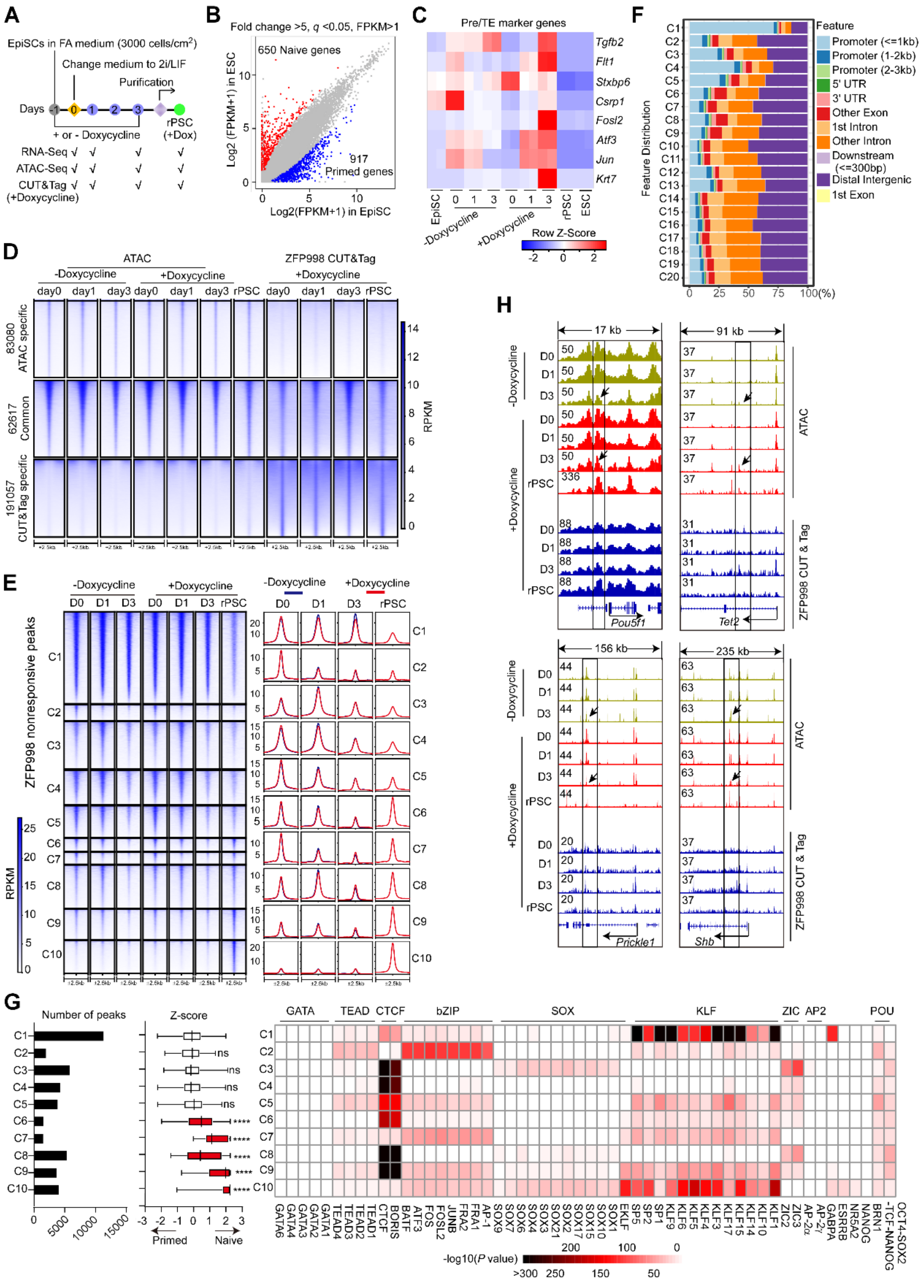

Supplemental Figure 11: ZFP998 binds to a set of enhancers and regulates their chromatin accessibility during primed-to-naïve reset.

(A) Experimental design for multi-omics sample collection. EpiSCs carrying a doxycycline-inducible ZFP998 transgene were treated with or without doxycycline (1 μg/mL) in 2i/LIF medium to initiate reprogramming. Cells were harvested for total

RNA (RNA-seq), native chromatin (ATAC-seq), and fixed chromatin (CUT&Tag for ZFP998, performed only in doxycycline-treated samples to detect induced protein binding) at the indicated time points (day 0, 1, 3). Established naïve PSCs and EpiSCs were included as reference points.

(B) Volcano plot of differentially expressed genes (DEGs) between EpiSCs and ESCs from RNA-seq data (two biological replicates per condition). DEGs were defined using Cuffdiff2 with thresholds of fold change > 5, $q < 0.05$, and FPKM > 1 in at least one condition. Naïve-enriched (n = 650) and primed-enriched (n = 917) gene sets are highlighted.

(C) Heatmap showing RNA-seq expression of previously defined primitive endoderm (PrE) and trophectoderm (TE) signature genes during the reset process with or without exogenous ZFP998 expression.

(D) Heatmap of ATAC-seq peaks and ZFP998 CUT&Tag peaks. Peaks were divided into three categories: ATAC-specific, ATAC- and CUT&Tag-common, and CUT&Tag-specific.

(E) Ten clusters of ATAC-seq peaks with accessibility not influenced by ZFP998 (ZFP998 nonresponsive peaks). Left panels show heatmaps of ATAC-seq accessibility, and right panels show pileups of the corresponding tag intensity.

(F) Bar plot showing the distribution of peaks from each cluster (defined in E and Figure 6C) across promoter (±2.5 kb from TSS), intronic, exonic, and intergenic regions.

(G) ZFP998-non-responsive peak categories and corresponding motif enrichment. Analysis is analogous to that in Fig. 6D, F. Left panel: number of peaks per cluster (defined in E). Middle panel: **r**elative expression of nearby naïve/primed genes (Z-score; significance versus C1 by Mann-Whitney U test: ****$P < 0.0001$; ns, not significant). Right panel: motif enrichment for transcription factors (color intensity represents $-\log_{10}$ (*P value*).

(H) Integrative Genomics Viewer (IGV) tracks showing ATAC-seq signal (normalized as RPKM) and ZFP998 CUT&Tag enrichment at enhancers associated with naïve-promoting (*Pou5f1*, *Tet2*) and primed (*Prickle1*, *Shb*) genes during the time course of reprogramming.

All experiments were performed with two independent biological replicates.

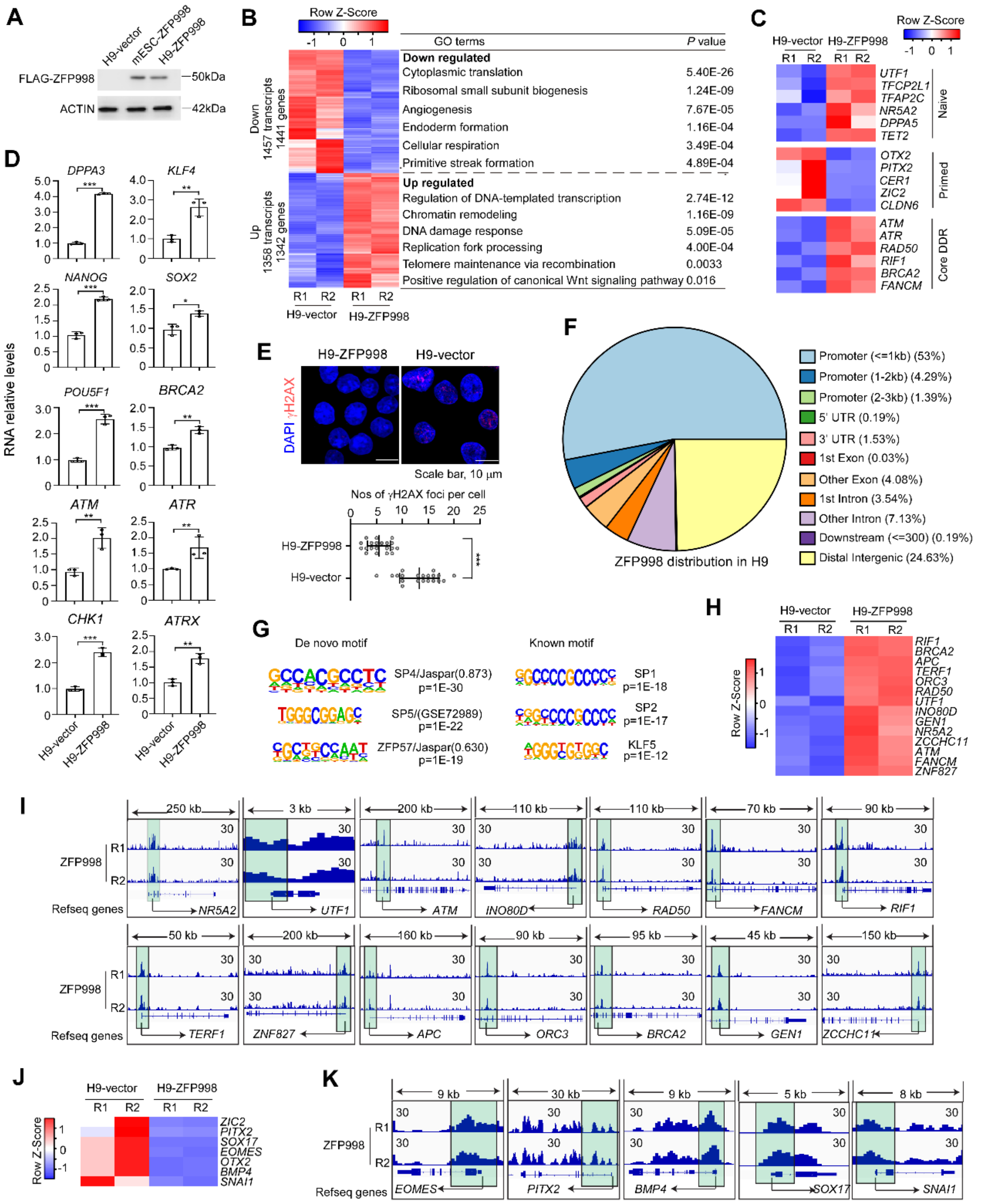

Supplemental Figure 12: Ectopic expression of ZFP998 in H9 hESCs up-regulates pluripotency and DDR genes.

(A) Western blot confirmed the ectopic expression of ZFP998 in H9 hESCs. Total protein lysates from H9 hESCs transduced with an empty vector control (H9-vector) or a vector expressing mouse ZFP998 (H9-ZFP998) were subjected to immunoblot analysis using anti-FLAG antibody (for the tagged transgene) and anti-ACTIN antibody (loading control). Three independent experiments were performed.

(B) DEGs between H9-vector and H9-ZFP998 hESCs cultured in E8 medium. Heatmap of DEGs are shown in the left panel (fold change > 1.5, $q < 0.05$). Corresponding GO enrichment terms are shown in the right panel.

(C) Heatmap of representative gene expression. A set of naïve-associated, primed-associated, and core DDR genes are shown for H9-vector and H9-ZFP998 hESCs (two biological replicates each).

(D) Increased expression of selected pluripotency (*DPPA3*, *NANOG*, *KLF4*, *SOX2*, *POU5F1*) and DDR (*BRCA2*, *ATM*, *ATR*, *CHEK1*, *ATRX*) genes in H9-ZFP998 ESCs was validated by quantitative RT-PCR. RNA expression was normalized to *ACTB*. H9-vector was set to 1. Data are presented as mean ± SEM from three independent RNA preparations and analyzed using two-tailed Student's *t*-test. *$P < 0.05$, **$P < 0.01$, ***$P < 0.001$.

(E) Immunostaining showed that H9-ZFP998 hESCs expressed lower levels of γH2AX compared with H9-vector hESCs. Nuclei were counterstained with DAPI. Representative images are shown in the top panel. Quantification of the mean number of γH2AX foci per nucleus is shown in the bottom panel. At least 30 fields of view containing 200 cells per condition were examined.

(F) Genomic distribution of ZFP998 binding sites in hESCs. Pie chart shows the percentage of ZFP998 CUT&Tag peaks annotated to promoter (± 2.5 kb from TSS), intronic, exonic, and intergenic regions.

(G) Motifs enrichment analysis of ZFP998 binding sites in hESCs was performed. The top enriched *de novo* motifs are shown in the left panel, and known motif enrichment is shown in the right panel.

(H and I) Examples of ZFP998-directly-upregulated genes. **(H)** Heatmap of representative genes whose expression increased in H9-ZFP998 cells and whose promoters are bound by ZFP998 is shown. **(I)** Integrative Genomics Viewer (IGV) tracks showing ZFP998 CUT&Tag signals at the corresponding genes from **(H)**.

(J and K) Examples of ZFP998-directly-downregulated genes. **(J)** Heatmap of RNA-seq expression of representative genes whose expression decreased in H9-ZFP998 cells and whose promoters are bound by ZFP998 is shown. **(K)** IGV tracks show ZFP998 CUT&Tag signals at the corresponding genes from **(J)**.
